# Bisphenol-A Impairs Insulin Action and Up-Regulates Inflammatory Pathways in Human Subcutaneous Adipocytes and 3T3-L1 Cells

**DOI:** 10.1371/journal.pone.0082099

**Published:** 2013-12-09

**Authors:** Rossella Valentino, Vittoria D’Esposito, Federica Passaretti, Antonietta Liotti, Serena Cabaro, Michele Longo, Giuseppe Perruolo, Francesco Oriente, Francesco Beguinot, Pietro Formisano

**Affiliations:** 1 Istituto di Endocrinologia ed Oncologia Sperimentale (IEOS-CNR), Naples, Italy; 2 Dipartimento di Scienze Mediche Traslazionali, Università degli Studi di Napoli “Federico II”, Naples, Italy; 3 Dipartimento di Scienze Farmaceutiche, Università degli Studi di Salerno, Salerno, Italy; Universidad Miguel Hernández de Elche, Spain

## Abstract

Current evidence indicates that chemical pollutants may interfere with the homeostatic control of nutrient metabolism, thereby contributing to the increased prevalence of metabolic disorders. Bisphenol-A (BPA) is a lipophilic compound contained in plastic which is considered a candidate for impairing energy and glucose metabolism. We have investigated the impact of low doses of BPA on adipocyte metabolic functions. Human adipocytes derived from subcutaneous adipose tissue and differentiated 3T3-L1 cells were incubated with BPA, in order to evaluate the effect on glucose utilization, insulin sensitivity and cytokine secretion. Treatment with 1nM BPA significantly inhibited insulin-stimulated glucose utilization, without grossly interfering with adipocyte differentiation. Accordingly, mRNA levels of the adipogenic markers PPARγ and GLUT4 were unchanged upon BPA exposure. BPA treatment also impaired insulin-activated receptor phosphorylation and signaling. Moreover, adipocyte incubation with BPA was accompanied by increased release of IL-6 and IFN-γ, as assessed by multiplex ELISA assays, and by activation of JNK, STAT3 and NFkB pathways. Treatment of the cells with the JNK inhibitor SP600125 almost fully reverted BPA effect on insulin signaling and glucose utilization. In conclusion, low doses of BPA interfere with inflammatory/insulin signaling pathways, leading to impairment of adipose cell function.

## Introduction

Overweight, obesity and insulin resistance epidemics are significant human health problems in adults, but also in children and adolescents [1], [2]. They are associated with increased risk of diseases related to metabolic dysfunctions, including metabolic syndrome (MS), type 2 diabetes mellitus (T2D), coronary heart disease (CHD), and some forms of cancer [3]–[6]. Chronic inflammation often accompanies obesity and related disorders, suggesting that inflammatory factors might be a crucial culprit connecting adipose tissue dysfunction with insulin resistance and diabetes as well as with cardiovascular disease and cancer [5]–[9].

Environmental signals, including chemical pollutants, are now considered contributing factors for insulin resistance [10]–[13]. Most of chemical pollutants potentially accumulate into adipose tissue [14], and could be responsible, in addition to the well-known dietary and lifestyle habits, for overweight and obesity epidemic, driving the parallel T2D epidemic [13], [15]–[17].

The “environmental obesogen hypothesis” has been associated to the adipose tissue inflammatory phenotype, with hyper-secretion of pro-inflammatory and decrease of anti-inflammatory cytokines, and considered as a major contributing factor for the decreased insulin sensitivity [7]–[10]. Indeed, the exponential rise of obesity-related pathologies worldwide is associated with the marked increase of toxic chemicals in the environment. In the modern world, in fact, the environment has been largely affected by an ever increasing number of synthetic chemical lipophilic pollutants, such as pesticides, organophosphates, polychlorinated bisphenyls, phthalates, solvents etc., that permeate the diet, the air and the ground [15], [16], [18]–[20]. An even larger impact could be exerted by those which are resistant to biological and chemical degradation, also termed Persistent Organic Pollutants(POPs), most of them also classified as endocrine disruptors [15]–[20].

Bisphenol-A (BPA) represents a potential obesogen compound and has been studied for its estrogen mimetic activity and endocrine disruption [21]–[24]. BPA is found in products containing polycarbonate plastics and in resins, as lining for metal cans and as an additive in other widely used plastics [25]–[27]. It is a lipophilic compound detectable at nanomolar levels not only in food and tap water, in rivers, lakes and sea, but also in human blood samples and urine worldwide, as well as in the placenta, amniotic fluid of pregnant women and in human milk [28], [29]. In animal models, BPA has been shown to disrupt the major weight controlling hormones, such as thyroid hormones, estrogens, testosterone, corticosteroids, growth hormone and leptin, and to alter adipogenesis, beta-cell and endocrine pancreas function [24], [30]–[32]. The relationship between BPA, inflammation and insulin sensitivity in adipose tissue is still not well understood.

Thus, we have investigated whether BPA, at nanomolar doses, consistent with those found in the human bloodstream and/or in environment, could interfere with adipose tissue function. We found that, in cultured adipose cells derived from human subcutaneous tissue and in 3T3-L1 adipocytes, BPA exposure impaired insulin sensitivity and glucose utilization and enhanced the release of pro-inflammatory compounds, even in the absence of major derangement of adipocyte differentiation.

## Materials and Methods

### Materials

Media, sera, and antibiotics for cell culture were from Lonza (Lonza Group Ltd, Basel, Switzerland). Antibodies against phospho-Ser_473_PKB/Akt1, ERK,phospho-Thr_183_/Tyr_185_c-Jun N-terminal kinase (JNK), STAT3, NFkB, Laminin A/C and actin were purchased from Santa Cruz Biotechnology (Santa Cruz, CA). Phospho-Thr_202_/Tyr_204_ERK, Phospho-IGF1-Receptor beta Tyr_1131_/Insulin Receptor beta Tyr_1146_, Insulin Receptor beta and Phospho-Tyr_705_ STAT3 antibodies were obtained from Cell Signaling Technology (Danvers, MA). PKB/Akt antibody was from Millipore (Billerica, MA). BPA in ethanol was a generous gift of Prof. C. Crescenzi (Department of Pharmaceutic Science, University of Salerno, Italy). Sodium dodecyl sulfate-polyacrylamide gel electrophoresis (SDS-PAGE) reagents were from Bio-Rad (Hercules, CA). All the other chemicals were from Sigma-Aldrich (St. Louis, MO).

### Cell cultures

Human adipose tissue samples were obtained by abdominal biopsy in the periumbilical region under local anesthesia (2% lidocaine) from patients undergoing elective abdominal surgery for gall bladder disease. The study was approved by the Ethic Committee of the University of Napoli “Federico II”. The written informed consent with respect to taking the samples and making the cell lines was obtained before the bioptical procedure. The study protocol was conducted in accordance to the principles of the Declaration of Helsinki as revised in 2000. Adipose tissue was digested with collagenase and Adipose-derived Stromal Vascular Fraction cells (SVF) were isolated and differentiated as previously reported [6].

table-1-caption3T3-L1 mouse embryonic fibroblasts (ATCC-CL-173, American Type Culture Collection, Manassas, VA) were cultured in Dulbecco’s modified Eagle’s medium (DMEM) supplemented with 10% fetal bovine serum (FBS) and 2 mmol/l glutamine, 100 IU/ml penicillin, 100 IU/ml streptomycin. Cultures were maintained in humidified atmosphere of 95% air and 5% CO_2_ at 37 C. 3T3-L1 differentiation has been achieved as previously described [6].

Conditioned media were obtained by incubating the cells for 8 h with serum-free DMEM 0.25% BSA after two washes with PBS. After the incubation, medium was collected and centrifuged at 14000g to remove cellular debris and analyzed for cytokine content, as described below.

### Glucose Utilization

table-1-captionFor glucose utilization studies, the method previously described [33] was modified for human adipocytes and 3T3-L1 adipocytes. Adipocytes were incubated in serum-free media containing 0.25% BSA in the absence or presence of 1 nM–100 nM BPA and 100 nM insulin for 24–48h. Glucose concentration was measured in the medium before and after the incubation.

table-1-captionThe difference in glucose concentration was considered to be utilized by the cells. Quantitative analysis of glucose concentration was performed with a ABX Pentra 400 clinical chemistry analyzer using the reagent ABX Pentra Glucose CP (ABX-Horiba, Montpellier, France) according to the manufacturer's instructions.

### Immunoblot procedure

Total cell lysates and, where indicated, cytosolic and nuclear fractions were obtained, separated by SDS-PAGE and immunoblotted with specific antibodies as previously described [34]–[35].

### Cytokine Assay

Human and 3T3-L1 adipocytes conditioned media were screened for the concentration of IL-1a, IL-1b, IL-2, IL-3, IL-4, IL-5, IL-6, IL-9, IL-10, IL-12 (p40), IL-12(p70), IL-13, IL-17, Eotaxin, G-CSF, GM-CSF, IFN-γ, KC/IL-8, MCP-1, MIP-1α, MIP-1β, RANTES and TNF-α using the Bioplex multiplex Mouse and Human Cytokine kits (Bio-Rad) according to the manufacturer’s protocol as previously described [6].

### Real-time RT-PCR analysis

table-1-captionTotal RNA was isolated from 3T3-L1 adipocytes by using the RNeasy Kit (Qiagen Sciences) according to the manufacturer’s instruction. For real-time RT-PCR analysis, 1 µg cell RNA was reverse transcribed using Super Script II Reverse Transcriptase (Invitrogen). PCR were analyzed using SYBR Green mix (Invitrogen). Reactions were performed using Platinum SYBR Green Quantitative PCR Super-UDG using an iCycler IQmulticolor Real-Time PCR Detection System (Bio-Rad). All reactions were performed in triplicate and β-actin was used as an internal standard. Primer sequences used were described in Table 1.

**Table 1:** Primer sequences used in Real-time RT-PCR analysis.

PPARγ Forward	5′- TGG TGC CTTCGCTCATGC -3′
PPARγ Reverse	5′- CTG TGG TAA AGG GCT TGA TGTC -3′
GLUT4 Forward	5′- CAG AAG GTG ATT GAA CAG AG- 3′
GLUT4 Reverse	5′- AAT GAT GCC AAT GAG AAA GG-3′
GLUT1 Forward	5′- GGG AAT GTC CTC ATC TTG GA -3′
GLUT1 Reverse	5′- TGA GGC TCT GTG TGG TTC TG -3′
LEPTIN Forward	5′-ACTCCACAATGCTTGACTC -3′
LEPTIN Reverse	5′-CCTACCTCACCTCTCCTG -3′
β-actin Forward	5′- CGC CCT AGG CAC CAG GGT GTG -3′
β-actin Reverse	5′- TCG GTG AGC AGC ACA GGG TG -3′

### Statistical analysis

Data were analyzed with Statview software (Abacus concepts) by one-factor analysis of variance. *p* values of less than 0.05 were considered statistically significant.

## Results

### BPA down-regulates insulin-stimulated glucose utilization in differentiated adipocytes

**Figure 1:**
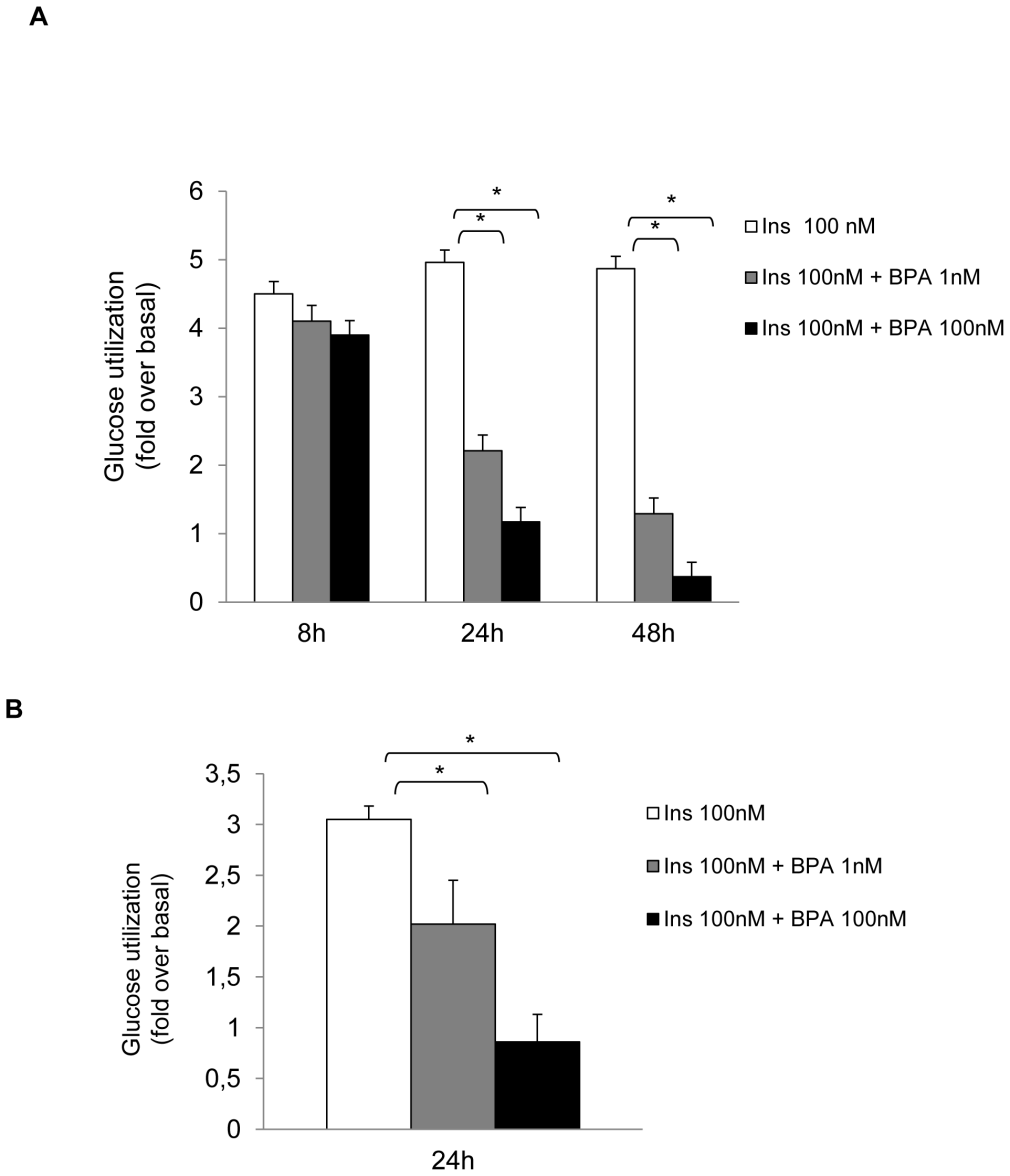
Effect of BPA on adipocyte glucose utilization. Human adipocytes (a) and 3T3-L1 adipocytes (b) were incubated in serum free-media with 1 nM or 100 nM BPA and 100 nM insulin for 24 and 48h as indicated. Next, supernatants were collected and glucose consumption was determined as described in Materials and Methods. Bars represent the mean ± SD of three independent experiments. Data were analyzed with Statview software (Abacus concepts) by one-factor analysis of variance. *p* values of less than 0.05 were considered statistically significant. Asterisks indicate statistically significant differences (* p<0.05). Error bars indicate mean ± S.D.

We have first investigated whether BPA could affect glucose utilization in differentiated adipocytes. To this end, human adipocytes, obtained following differentiation of adipose tissue-derived stromal vascular fraction (SVF) cells, were incubated with BPA (1 nM or 100 nM), for 8, 24 and 48h, in the absence or in the presence of insulin (100 nM). Following BPA treatment, glucose utilization tended to be increased, at each time point (data not shown). As expected, in the absence of BPA, insulin induced a significant 4.5 fold increase of glucose utilization. Insulin stimulatory effect was still detectable after 8h incubation with 1 nM BPA, while after 24h of incubation with BPA, insulin effect on glucose utilization was reduced by 55% (Fig 1a).Within the same time frame, 100 nM BPA decreased insulin stimulatory action by >70%. Prolonging BPA pre-exposure to 48h, further reduced insulin effect. Consistent results were obtained following identical treatment of differentiated 3T3-L1 adipocytes (Fig 1b). Thus, 1 nM BPA treatment for 24h was sufficient to inhibit insulin effect on glucose utilization both in differentiated human adipocytes and in 3T3-L1 cells. Therefore, for the next experimental settings we have used 1 nM BPA, as the lowest non toxic dose.

No relevant morphological abnormalities were observed following BPA exposure (up to 48 h) both for human and 3T3-L1 adipocytes.

**Figure 2:**
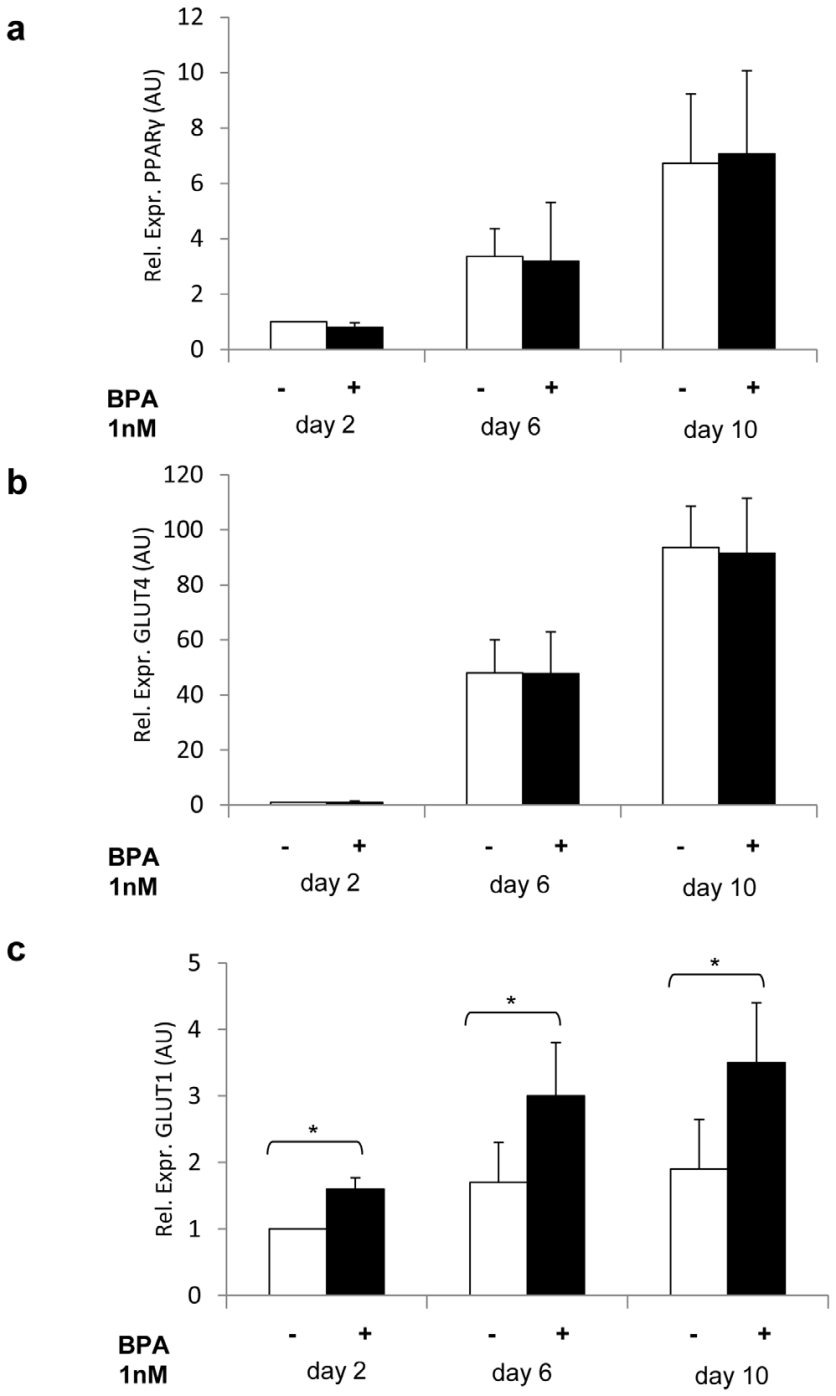
Effect of BPA on adipocyte gene expression. 3T3-L1 cells have been differentiated in mature adipocytes in presence of BPA 1 nM. Next, mRNA levels of PPARγ (a), GLUT4 (b) and GLUT1 (c) during adipogenic differentiation were determined by real-time RT-PCR analysis. Bars represent the mean ± SD of four independent experiments and show the mRNA levels in these cells relative to those in 3T3-L1 cells in absence of BPA at day 2 of differentiation. Data were analyzed with Statview software (Abacus concepts) by one-factor analysis of variance. *p* values of less than 0.05 were considered statistically significant. Asterisks indicate statistically significant differences (* p<0.05). Error bars indicate mean ± S.D.

Moreover, 1 nM BPA did not roughly affect adipocyte differentiation. Indeed, when 3T3-L1 pre-adipocytes were incubated with BPA along with the differentiation mix, during early (day 2), medium (day 6) and late (day 10) adipogenesis phases, PPARγ (Fig.2a) and GLUT4 (Fig.2b) mRNA levels were unchanged. At variance, GLUT1 levels were increased, following BPA exposure (Fig.2c). Consistent results were obtained with human pre-adipocytes (data not shown).

### BPA inhibits insulin receptor phosphorylation and downstream signalling in differentiated adipocytes

**Figure 3:**
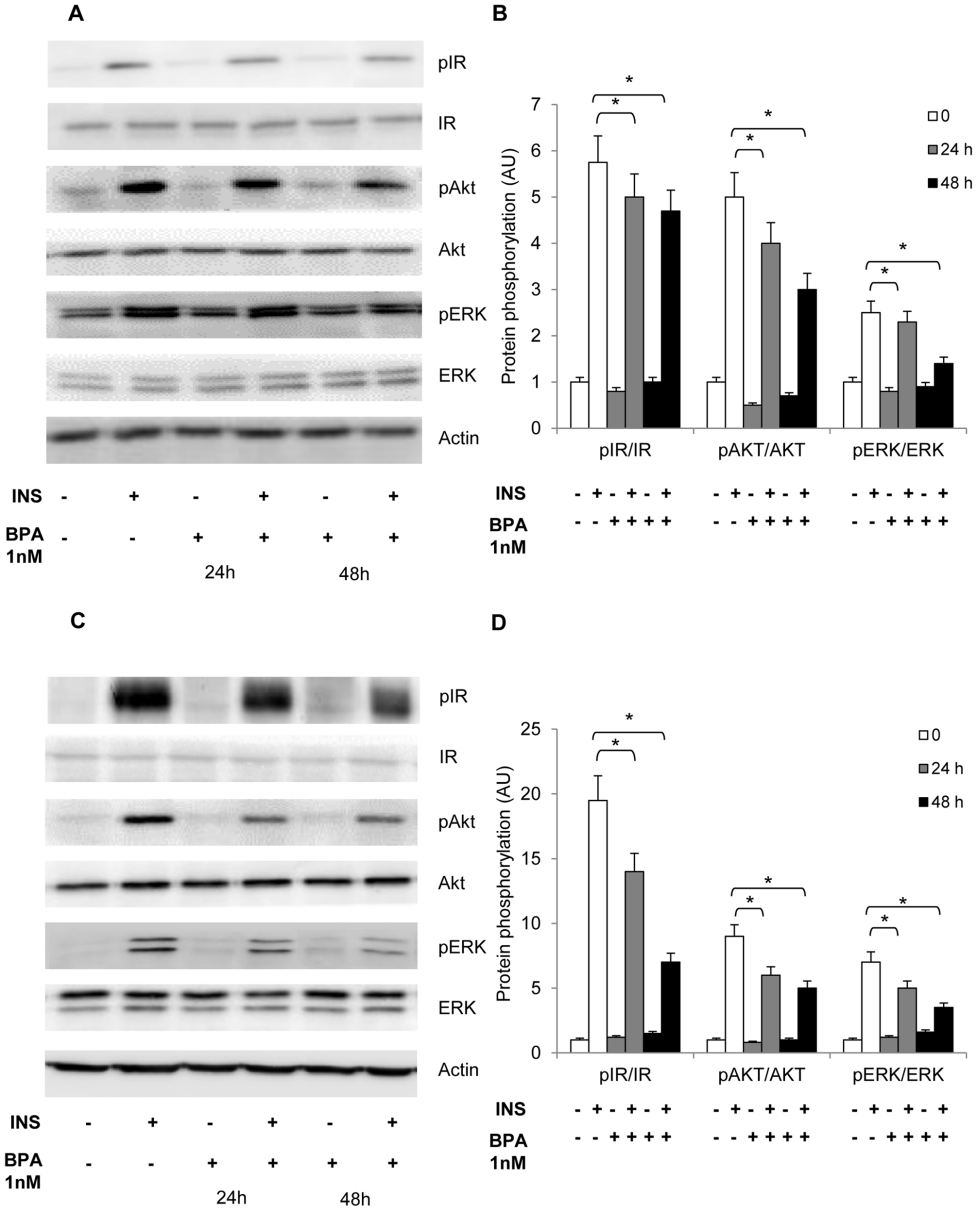
Effect of BPA on insulin transduction pathways. Human (a) and 3T3-L1 adipocytes (c) were incubated with 1 nM BPA for 24 and 48h as indicated and exposed to 100 nM insulin for 10 min and then solubilized as described in Materials and Methods. Cell lysates (50 µg protein/sample) were blotted with phospho- Tyr_1146_ Insulin Receptor β (pIR), phospho- Ser_473_Akt/PKB and phospho-Thr_202_/Tyr_204_Extracellular signal-Regulated Kinases (pERK) antibodies and then reblotted with anti-IR, Akt/PKB and ERK antibodies. To ensure the equal protein transfer, membranes were blotted with actin antibodies. The filters were revealed by ECL and autoradiography. The autoradiographs shown are representative of four independent experiments. b-d) Filters obtained in *a* and *c* have been analyzed by laser densitometry as described under Materials and Methods. Data were analyzed with Statview software (Abacus concepts) by one-factor analysis of variance. *p* values of less than 0.05 were considered statistically significant. Asterisks indicate statistically significant differences (*p< 0.05). Error bars indicate mean ± S.D.

We have therefore analyzed the effect of BPA on insulin signalling in mature adipocytes. Human (Fig. 3a-b) and 3T3-L1 (Fig.3c-d) adipocytes were pre-incubated with 1 nM BPA for 24h and 48h and then stimulated with insulin for 10 min. BPA exposure led to a time-dependent decrease of insulin-stimulated insulin receptor (IR) tyrosine phosphorylation and to a significant reduction of PKB/Akt and ERK1/2phosphorylation. No significant BPA-dependent change was observed for IR, PKB/Akt and ERK1/2 total protein or basal phosphorylation levels.

### BPA exposure affects adipocyte secretion and induces activation of inflammation-related pathways in adipocytes

**Figure 4:**
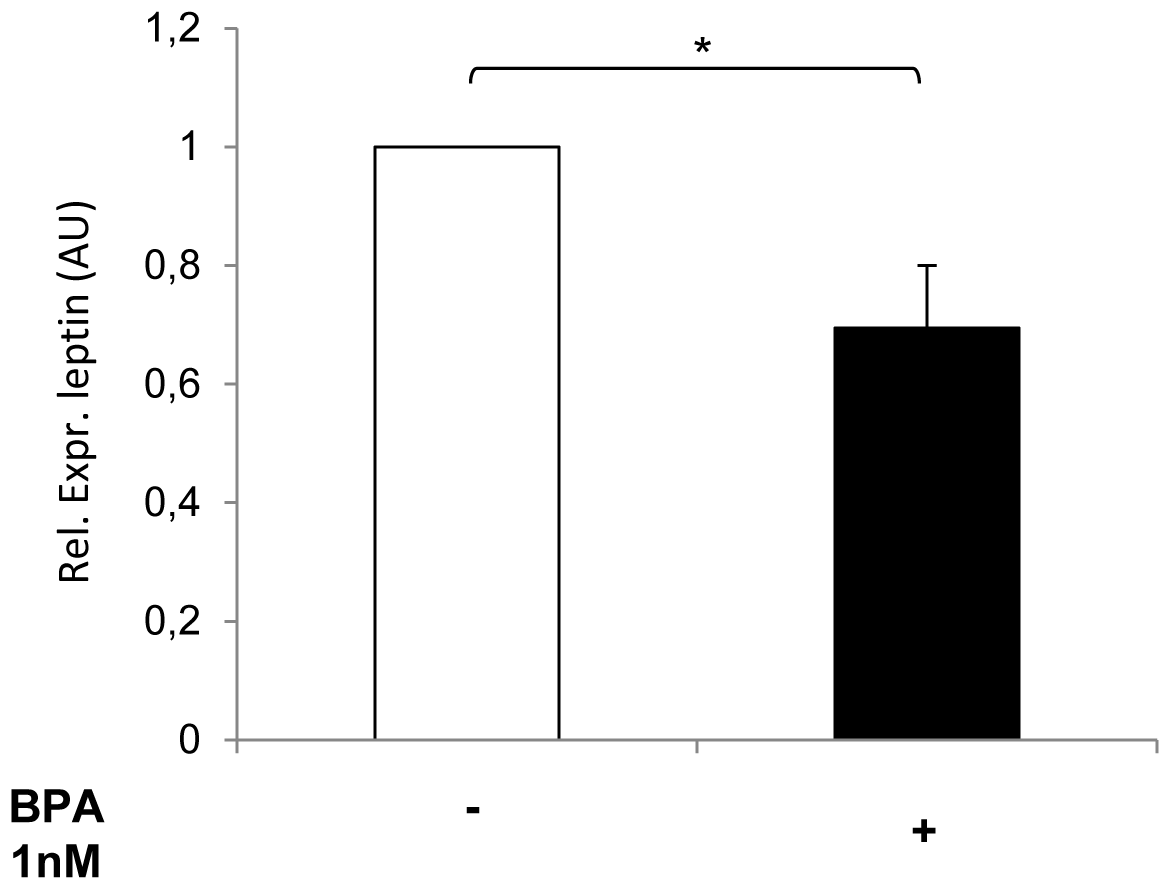
Effect of BPA on leptin mRNA levels. 3T3-L1 adipocytes were incubated with 1 nM BPA for 24h and leptin mRNA levels were determined by real-time RT-PCR analysis. Bars represent the mean ± SD of three independent experiments and show the leptin mRNA levels relative to those in 3T3-L1 cells in absence of BPA. Data were analyzed with Statview software (Abacus concepts) by one-factor analysis of variance. *p* values of less than 0.05 were considered statistically significant. Asterisks indicate statistically significant differences (*p< 0.05). Error bars indicate mean ± S.D.

We assessed whether BPA could affect the expression of specific adipokines. To this end, we have measured leptin mRNA levels in 3T3-L1 cells. A significant decrease in leptin mRNA levels was found following treatment with 1 nM BPA, compared the control cells (Fig.4).

We have next analyzed the impact of 1 nM BPA on adipocyte secretory function, in terms of release of inflammatory cytokines (Table 2). Both human and 3T3-L1 differentiated adipocytes displayed higher levels of IL-6 and IFN-γ. No significant difference was found for several other cytokines (IL-1b, IL-2, IL-4, IL-6, IL-10, GM-CSF, IFN-γ, KC/IL-8, MIP-1α, MIP-1β, RANTES and TNF-α) with the exception of MIP-1α, which was significantly elevated in BPA-treated 3T3-L1, while not in human adipocytes. Some other cytokines (IL-1a, IL-3 IL-5, IL-9, IL-12 p40, IL-12p70, IL-13, IL-17, Eotaxin, G-CSF, MCP-1) were not detected in the conditioned media of either human and 3T3-L1 differentiated adipocytes.

**Table 2:** BPA interference on adipocyte-released cytokines.

	Human Adipo (pg/ml)	Human Adipo + BPA (pg/ml)	Adipo3T3-L1(pg/ml)	Adipo3T3-L1 +BPA (pg/ml)
**IL-1b**	**ND**	**ND**	**60.58±9**	**55.36±7.4**
**IL-2**	**9.63±1.2**	**9.68±0.8**	**ND**	**ND**
**IL-4**	**8.23±0.9**	**8.55±0.9**	**0.13±0.02**	**0.9±0.008**
**IL-6**	**689.7±72.5**	884.9±93.4*	**0.98±0.03**	5.27±0.7**
**IL-10**	**85.01±9.2**	**83.15±9.7**	**3.70±0.4**	**4.39±0.8**
**GM-CSF**	**112.31±15.6**	**114.41±13.1**	**ND**	**ND**
**IFN-γ**	**393.79±42.6**	481.71±45.7*	**0.25±0.09**	0.86±0.05*
**KC/IL-8**	**56.81±6.9**	**63.63±6.1**	**20566±215**	**21283±200.3**
**MIP-1a**	**2.44±0.9**	**2.52±0.7**	**26.22±3.2**	43.48±5.6*
**MIP-1b**	**13.68±1.9**	**14.07±2**	**2.35±0.52**	**1.26±0.3**
**RANTES**	**25.08±3.1**	**25.94±2.3**	**362.75±39.2**	**333.3±27.9**
**TNF-α**	**11.12±1.3**	**11.37±1**	**255.91±27.1**	**222.07±21.9**

Supernatants from 3T3-L1 and human adipocytes treated with or without 1 nM BPA for 24h were collected and tested by using the Bioplex multiplex cytokine assay kit as described in Materials and Methods. Data were analyzed with Statview software (Abacus concepts) by one-factor analysis of variance. *p* values of less than 0.05 were considered statistically significant. Asterisks indicate statistically significant differences (* p<0.05; ** p<0.01).

**Figure 5:**
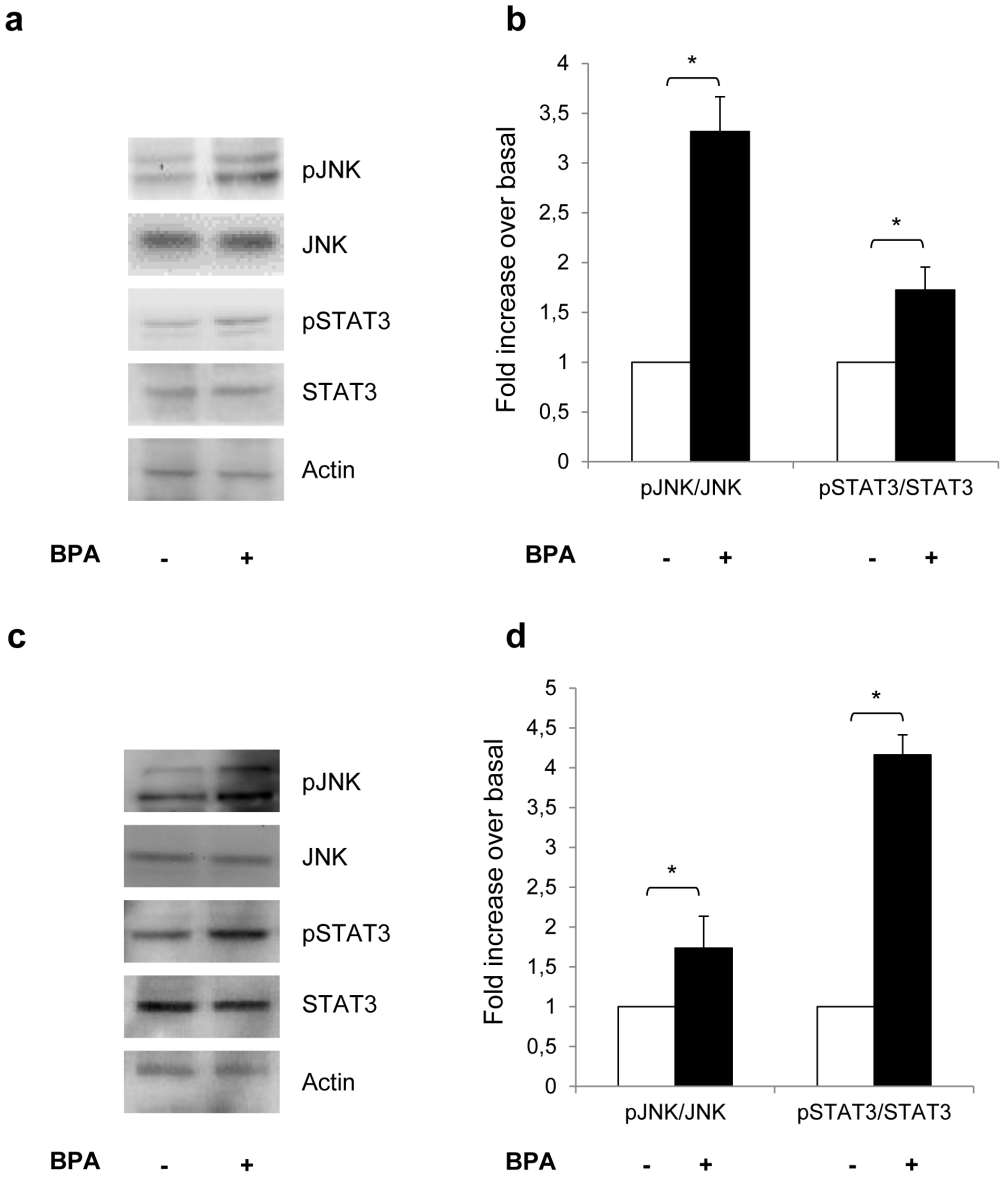
Effect of BPA on JNK and STAT3 activation. Human (a) and 3T3-L1 (c) adipocytes were incubated with 1 nM BPA for 24h and then solubilized as described in Materials and Methods. Cell lysates (50 µg protein/sample) were blotted with phospho- Thr_183_/Tyr_185_ JNK and Phospho-Tyr_705_ STAT3 antibodies and then reblotted with anti-JNK and STAT3 antibodies. To ensure the equal protein transfer, membranes were blotted with actin antibodies. The filters were revealed by ECL and autoradiography. The autoradiographs shown are representative of four independent experiments. b-d) Filters obtained in *a* and *c* have been analyzed by laser densitometry as described under Materials and Methods. Data were analyzed with Statview software (Abacus concepts) by one-factor analysis of variance. *p* values of less than 0.05 were considered statistically significant. Asterisks indicate statistically significant differences (*p<0.05). Error bars indicate mean ± S.D.

This led us to hypothesize that BPA may elicit an inflammatory-like response in adipocytes. We also observed a significantly increased detection of the phosphorylated forms of JNK and STAT3 in human (Fig. 5a-b) and 3T3-L1 (Fig. 5c-d) adipocytes upon treatment with BPA for 24h.

**Figure 6:**
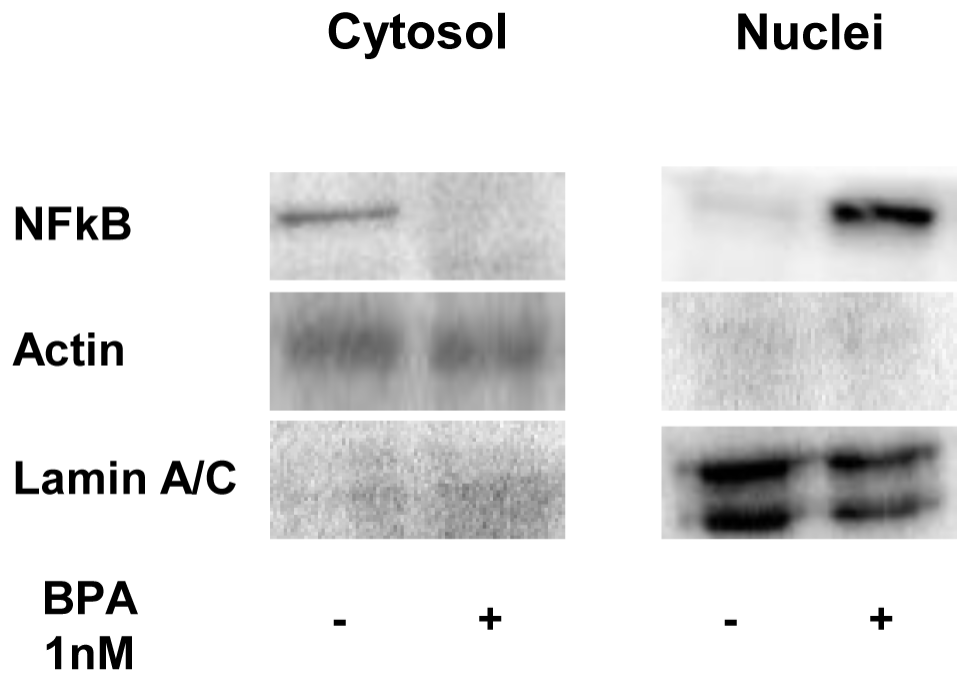
Effect of BPA on NFkB activation. 3T3-L1 adipocytes were incubated with 1 nM BPA for 24h. Next, subcellular fractionation(nuclear and cytoplasmic) was performed and proteins were extracted and subjected to SDS–PAGE and immunoblotted with anti-NFKB antibody. Actin and Laminin A/C were used as loading control for the cytosolic and nuclear fractions, respectively. Blots were revealed by ECL and autoradiography. The autoradiographs shown are representative of four independent experiments.

Consistently, 1 nM BPA treatment led to a significantly increased detection of NF-kB in nuclear extracts, paralleled by a decreased cytosolic abundance (Fig.6).

**Figure 7:**
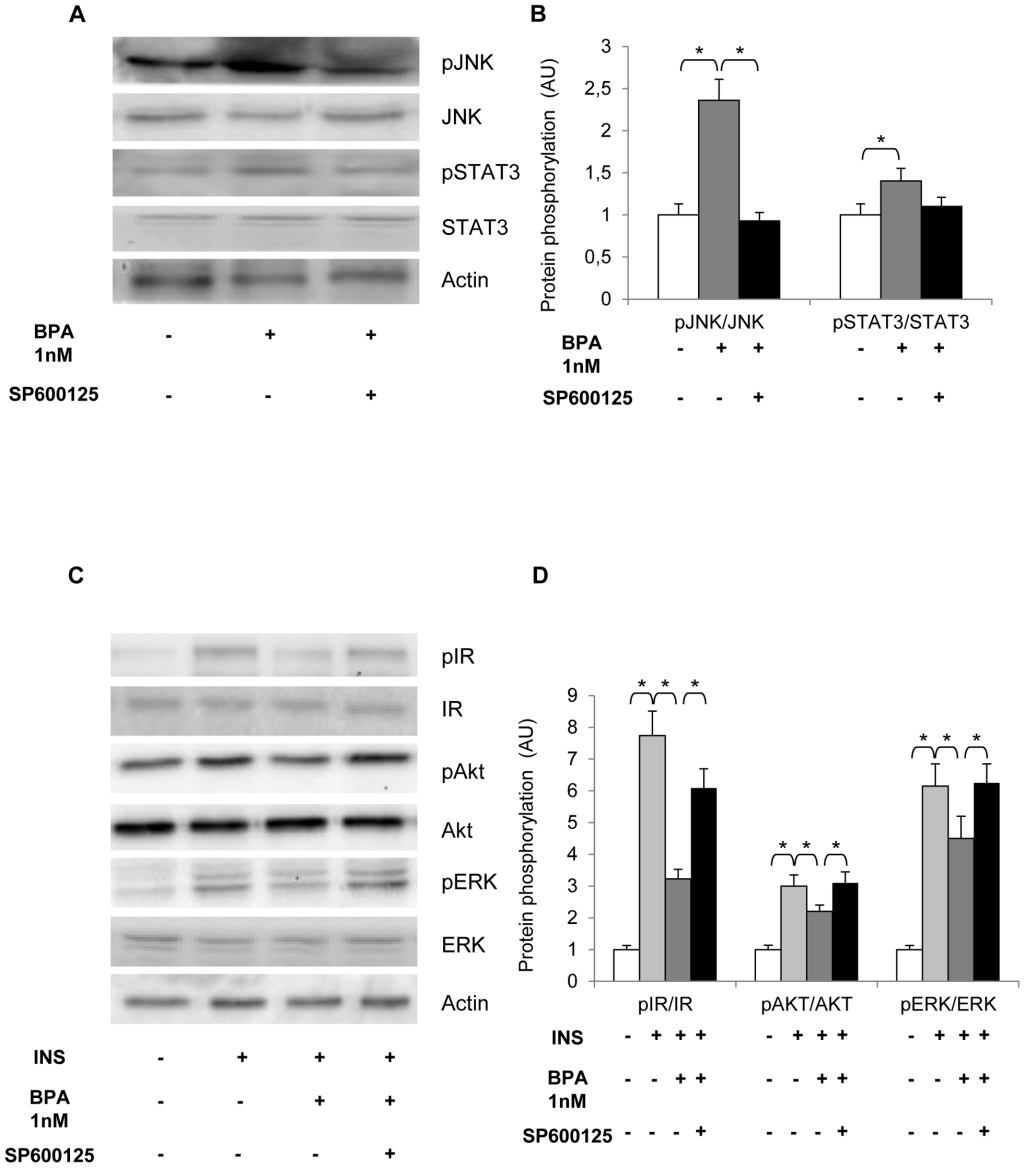
Effect of JAK2/STAT3 and JNK inhibition on BPA-impaired inflammatory and insulin pathways. Human adipocytes were incubated with 1 nM BPA for 24h and exposed to 20 µM SP600125 for 1 h. a) Cell lysates (50 µg protein/sample) were blotted with phospho-JNK and Phospho-Tyr_705_ STAT3 antibodies and then reblotted with anti- JNK and STAT3 antibodies. c) Cells were treated with 100 nM insulin for 10 min and then solubilized. Cell lysates (50 µg protein/sample) were blotted with phospho- IR, phospho- Ser_473_Akt/PKB and phospho-Thr_202_/ERK and then reblotted with anti-IR, Akt/PKB and ERK antibodies. To ensure the equal protein transfer, membranes were blotted with actin antibodies. The filters were revealed by ECL and autoradiography. The autoradiographs shown are representative of four independent experiments. b-d) Filters obtained in *a* and *c* have been analyzed by laser densitometry as described under Materials and Methods. Data were analyzed with Statview software (Abacus concepts) by one-factor analysis of variance. *p* values of less than 0.05 were considered statistically significant. Asterisks indicate statistically significant differences (* *p*<0.05). Error bars indicate mean± S.D.

**Figure 8:**
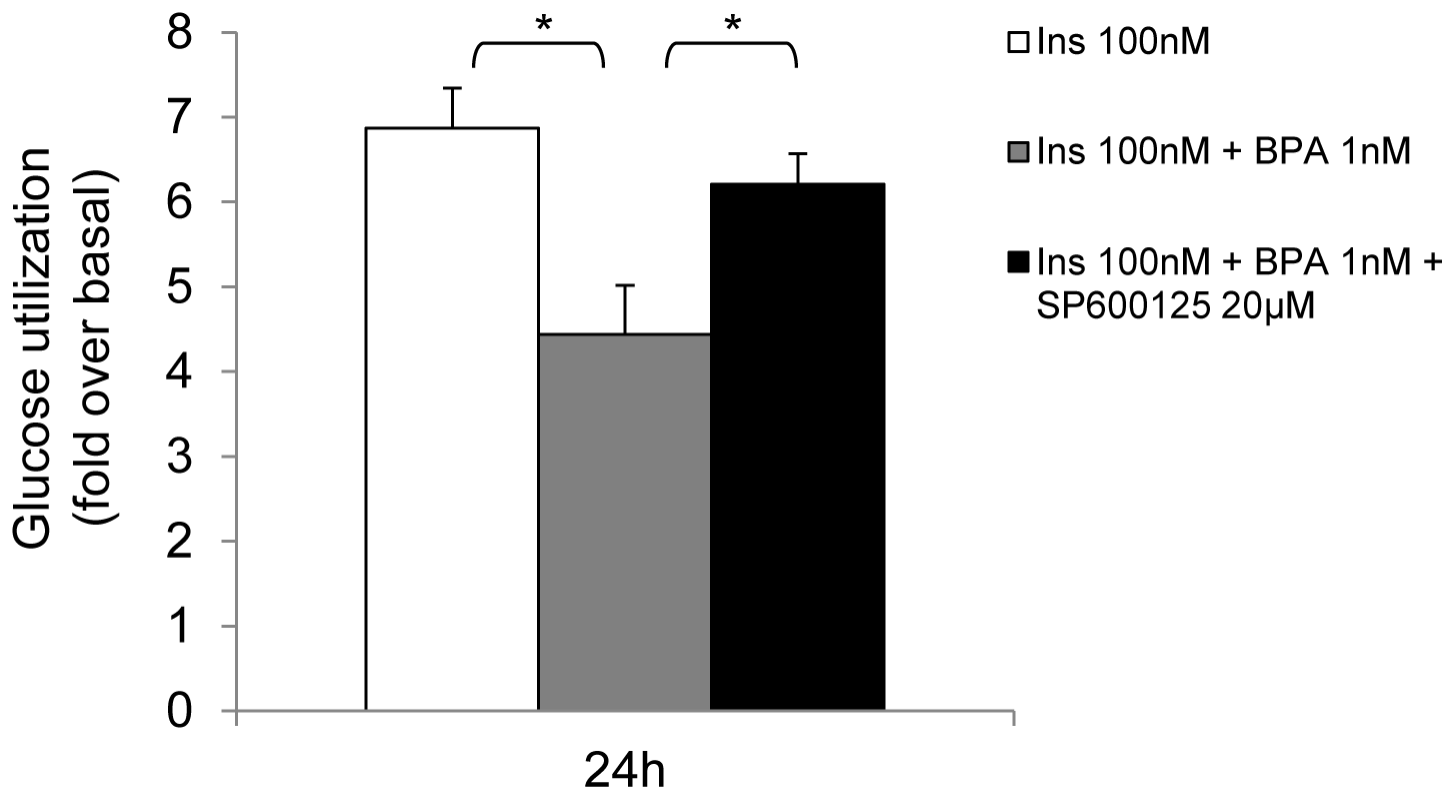
Effect of JAK2/STAT3 and JNK inhibition on BPA-impaired glucose utilization. Human adipocytes were incubated in serum free-media with 1 nM BPA, 20 µM SP600125 with or without 100 nM insulin for 24h as indicated. Next, supernatants were collected and glucose consumption was determined as described in Materials and Methods.Bars represent the mean ± SD of three independent experiments. Data were analyzed with Statview software (Abacus concepts) by one-factor analysis of variance. *p* values of less than 0.05 were considered statistically significant. Asterisks indicate statistically significant differences (* p<0.05). Error bars indicate mean± S.D.

Next, human adipocytes were treated with 20µM SP600125. At this concentration the compound inhibited BPA-stimulated JNK phosphorylation (Fig. 7a-b). We also observed a slight inhibition of STAT3 phosphorylation, however. Interestingly, the treatment with SP600125 largely rescued insulin effect on IR, PKB/Akt and ERK1/2 phosphorylation in BPA-stimulated cells (Fig.7c-d). We therefore tested the effect of SP600125 on glucose utilization on BPA-and insulin-stimulated cells (Fig. 8). Consistent with the effect on insulin signalling pathway, the compound reverted BPA inhibitory effect. Indeed, in the presence of SP600125 insulin action appeared inhibited by only 30%, compared to 55% inhibition occurring after BPA treatment alone.

## Discussion

Many environmental pollutants, including BPA, are lipophilic compounds with small size and with the ability to mimic or block the natural action of endogenous hormones [26]. For this capability, they may affect several endogenous pathways, including those regulating energy and glucose metabolism, thereby contributing to insulin resistance and metabolic dysfunction [36]

Moreover, environmental pollutants, as well as excessive nutrients, can trigger inflammatory signals. There is a large body of evidence indicating that obesity is accompanied by inflammation in metabolically relevant tissues, particularly in adipose tissue [7]–[10], [37]. It is therefore possible that pollutants may participate in deranging the function of adipose tissue, contributing to the inflammatory condition and to insulin resistance.

We have now provided evidence that nanomolar BPA concentrations may induce an inflammation-like response in human adipocytes. BPA exposure of human adipocytes increases the release of inflammatory factors, such as IL-6 and IFN-γ, raising the possibility that it may directly elicit a pseudo-inflammatory response in adipose tissue. Another consistent finding is the activation of typical inflammatory pathways. Indeed, JNK, JAK/STAT and NF-kB pathways have been found activated in BPA-treated adipocytes. Thus, both secretion of cytokines and activation of intracellular pathways, suggestive of an inflammatory response, occur after treatment with very low doses of BPA, comparable to those commonly found in the environment and in biological samples [28], [29]. The direct stimulation of BPA on inflammatory pathways in adipocytes could also be accompanied, in vivo, by an effect on inflammatory infiltrates in the adipose tissue [8]–[10], [38].

It has been hypothesized that BPA can trigger Toll-like Receptors (TLR), which in turn may induce JNK and NF-kB pathways, leading to up-regulation of pro-inflammatory factors, including IL-6 and IFN-γ [38], [39]. Alternatively, BPA may act through estrogen receptors both at genomic and at non-genomic level [39], [40]. Recent evidence also indicates that BPA specifically binds G protein-coupled receptor 30 (GPR30), a novel non-classical membrane ER, by which estrogenic compounds might induce biological effects in different cell types, including adipocytes [39], [41], [42].

Thus, one might speculate that BPA elicits intracellular signalling pathways (either via TLR, ER or GPR30) leading to up-regulation of IL-6 and IFN-γ, which in turn may contribute to activate typical inflammatory pathways, such as JNK and JAK/STAT. Consistent with our findings, other groups have shown that IL-6 release is increased following BPA exposure of human pre-adipocytes [39], [43]. To the best of our knowledge, this is the first report of IFN-γ release elicited by BPA in adipocytes, while it has been described in dendritic cells and in CD4^+^ lymphocytes [39], [43].

In parallel with the pseudo-inflammatory profile, we observed that BPA-treated adipocytes were less sensitive to insulin in terms of glucose utilization. Interestingly, the effect was already detectable upon treatment of the cells with 1 nM BPA. At this concentration, we failed to observe any gross abnormality in adipocyte morphology and differentiation markers, including GLUT4, suggesting that the alteration specifically occurred at the level of the insulin signalling machinery. Other laboratories have reported that BPA stimulated an increase in GLUT4 and glucose uptake in adipocytic cell models [44]. However, these *in vitro* effects required doses considerably higher than those found in human tissues [28]. Nonetheless, we observed increased basal glucose utilization, accompanied by increased levels of GLUT1. The possibility of reduced total glucose uptake is, therefore, not supported by data. At variance, we observed reduced insulin-stimulated tyrosine phosphorylation of insulin receptor and a consistent reduction of downstream signalling. In agreement with Kidani et al [45], these alterations can be responsible for a worsening in insulin signalling via PKB/Akt and ERK, causing a reduction in insulin sensitivity and in insulin-mediated glucose uptake in adipose tissue. This also raises the possibility that BPA-induced insulin sensitivity may contribute to worsen the pro-inflammatory profile. Indeed, an emerging body of evidence suggests that insulin suppresses the inflammatory process, not only through preventing hyperglycemia but also by modulating key inflammatory molecules [46]. Moreover, the decrease of leptin levels observed in BPA-treated adipocytes may be due to reduced insulin promotion of leptin gene expression, as previously reported in human adipocytes [47], [48].

Interestingly, however, inhibition of JNK activity almost completely restored insulin receptor signalling and largely rescued insulin-stimulated glucose utilization in BPA-treated adipocytes, suggesting a primary involvement of inflammatory factors.

Although no gross effect on adipocyte morphology and differentiation was observed, we cannot exclude the possibility that pre-natal or early post-natal exposure to BPA may affect adipogenesis as well, as has also been reported [49]–[53].

Thus, exposure to BPA impairs insulin sensitivity and induces the release of inflammatory factors in adipocytes. One possible mechanism is that BPA activates JNK, via TLRs or ERs, and this may directly impair insulin action [39], [54]. Alternatively, the release of IL-6 and IFN-γ may, in turn, contribute to JNK activation in the adipocytes and either directly or indirectly down-regulate insulin-stimulated glucose uptake [54].

It should be also pointed out that an associations between serum and urinary concentrations of persistent organic pollutants and diabetes have been described [13], [55], [56]. Moreover, we have recently reported that women with Polycystic Ovary Syndrome (PCOS), characterized by insulin resistance and low-grade chronic inflammation, displayed elevated serum BPA levels [57]. These observations indicate a relevant role for BPA in impairing energy and nutrient metabolism, and, together with our current report, suggest that adipocytes may integrate BPA signals and further derange insulin sensitivity and inflammatory pathways.
