# Correction: Bisphenol-A Impairs Insulin Action and Up-Regulates Inflammatory Pathways in Human Subcutaneous Adipocytes and 3T3-L1 Cells

**DOI:** 10.1371/journal.pone.0264656

**Published:** 2022-02-24

**Authors:** Rossella Valentino, Vittoria D’Esposito, Federica Passaretti, Antonietta Liotti, Serena Cabaro, Michele Longo, Giuseppe Perruolo, Francesco Oriente, Francesco Beguinot, Pietro Formisano

After publication of this article [1], concerns were raised about similarities between the Akt results shown in lanes 2–5 of Figure 3C and the Akt results shown in Fig 7C.

The authors provided underlying image data for all panels shown in Figs 3 and 7 (S1–S4 Files). The Fig 7C Akt panel appears to align with the raw image for the Figure 3C Akt experiment (lanes 3–6 of the Akt blot in S2 File) suggesting that an error was made in preparing Fig 7C. The authors provided raw images from experimental replicates in support of Fig 7C results (S4 and S5 Files), as well as an updated Fig 7 including the correct Akt data. S5 File includes additional raw images, including different exposures of the original blots and images in which lanes included in the published figures are marked for easy reference.

The raw image data revealed that the pAkt and pERK blot images were spliced to remove data between lanes 4 and 5 on the published Figure 3C (see S2 and S5 Files).

In regard to the control panels included in Figs 3 and 7, the authors clarified that the images included are representative examples from a full cohort of experimental results that included duplicates of each experiment. As such, the total protein, phosphoprotein, and β-actin controls shown in the Figures may not correspond to the same experiment and/or samples. For each experiment involving phospho-protein, control blots with β-actin and total protein were used. Each replicate experiment used independently prepared protein samples.

The raw data underlying other results in this article are available from the authors.

The authors apologize for the errors in the published article.

**Fig 7:**
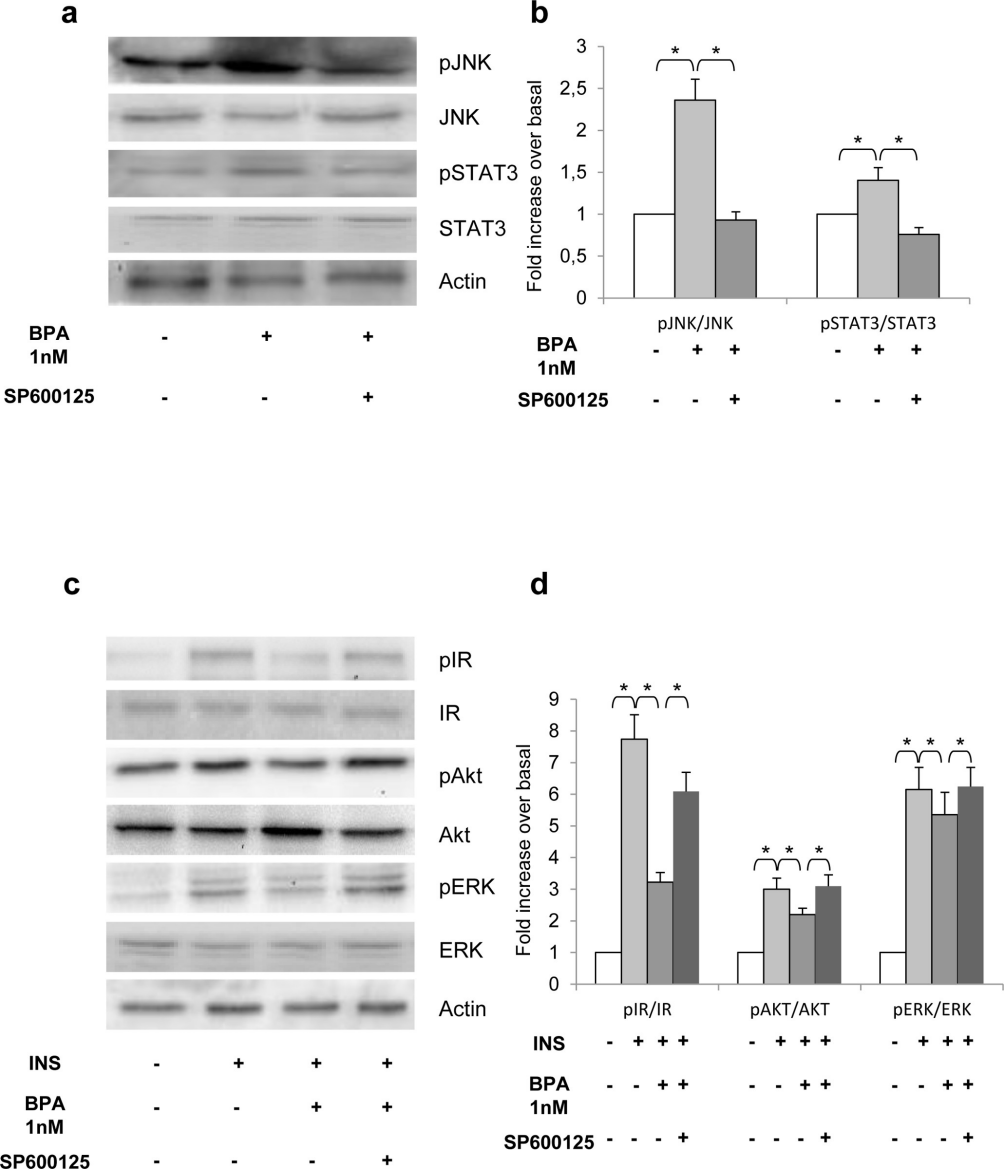
Effect of JAK2/STAT3 and JNK inhibition on BPA-impaired inflammatory and insulin pathways. Human adipocytes were incubated with 1 nM BPA for 24h and exposed to 20 μM SP600125 for 1 h. a) Cell lysates (50 μg protein/sample) were blotted with phospho-JNK and Phospho-Tyr_705_ STAT3 antibodies and then reblotted with anti- JNK and STAT3 antibodies. c) Cells were treated with 100 nM insulin for 10 min and then solubilized. Cell lysates (50 μg protein/sample) were blotted with phospho- IR, phospho- Ser_473_Akt/PKB and phospho-Thr_202_/ERK and then reblotted with anti-IR, Akt/PKB and ERK antibodies. To ensure the equal protein transfer, membranes were blotted with actin antibodies. The filters were revealed by ECL and autoradiography. The autoradiographs shown are representative of four independent experiments. b-d) Filters obtained in *a* and *c* have been analyzed by laser densitometry as described under Materials and Methods. Data were analyzed with Statview software (Abacus concepts) by one-factor analysis of variance. *p* values of less than 0.05 were considered statistically significant. Asterisks indicate statistically significant differences (* *p*<0.05). Error bars indicate mean± S.D.

## Supporting information

S1 File [S1.pdf]. Original blot data supporting the results in Figure 3A.
(PDF)

S2 File [S2.pdf]. Original blot data supporting the results in Figure 3C.
(PDF)

S3 File [S3.pdf]. Original blot data supporting the results in Fig 7A.
(PDF)

S4 File [S4.pdf]. Original blot data supporting the results in the corrected Fig 7C.
(PDF)

S5 File [follow-up question file.pdf]. Additional original blot data supporting the results in Figs 3 and 7.
(PDF)
