# Supplementary figures and images for: Correction: Bisphenol-A Impairs Insulin Action and Up-Regulates Inflammatory Pathways in Human Subcutaneous Adipocytes and 3T3-L1 Cells
Source: PLoS One. 2022 Feb 24;17(2):e0264656. doi: 10.1371/journal.pone.0264656 (PMC8870594; doi:10.1371/journal.pone.0264656)

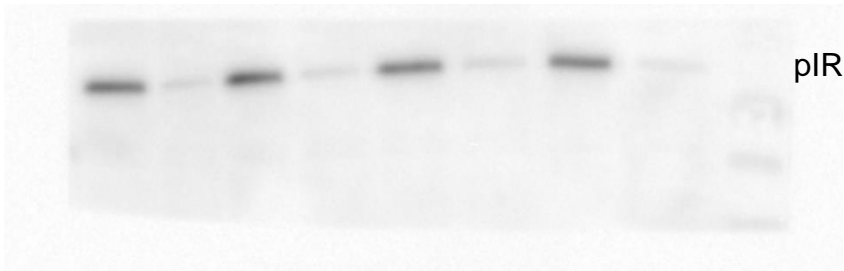

|            |   |   |     |   |     |   |
|------------|---|---|-----|---|-----|---|
| INS        | - | + | -   | + | -   | + |
| BPA<br>1nM | - | - | +   | + | +   | + |
|            |   |   | 24h |   | 48h |   |

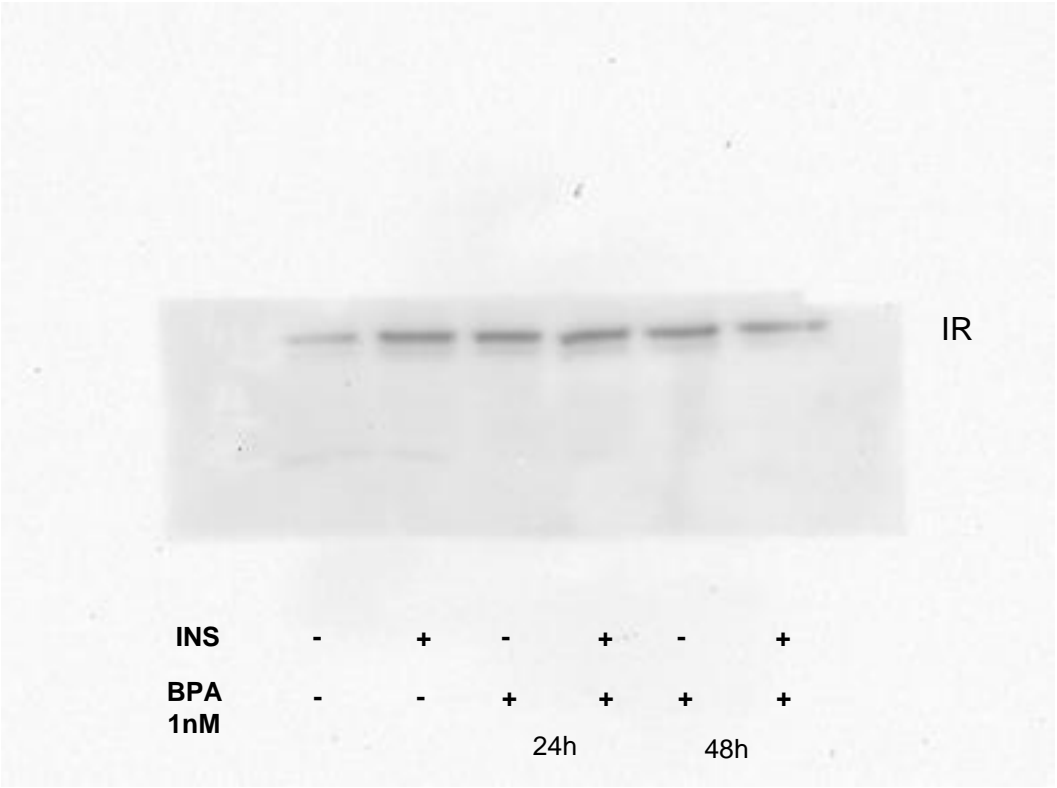

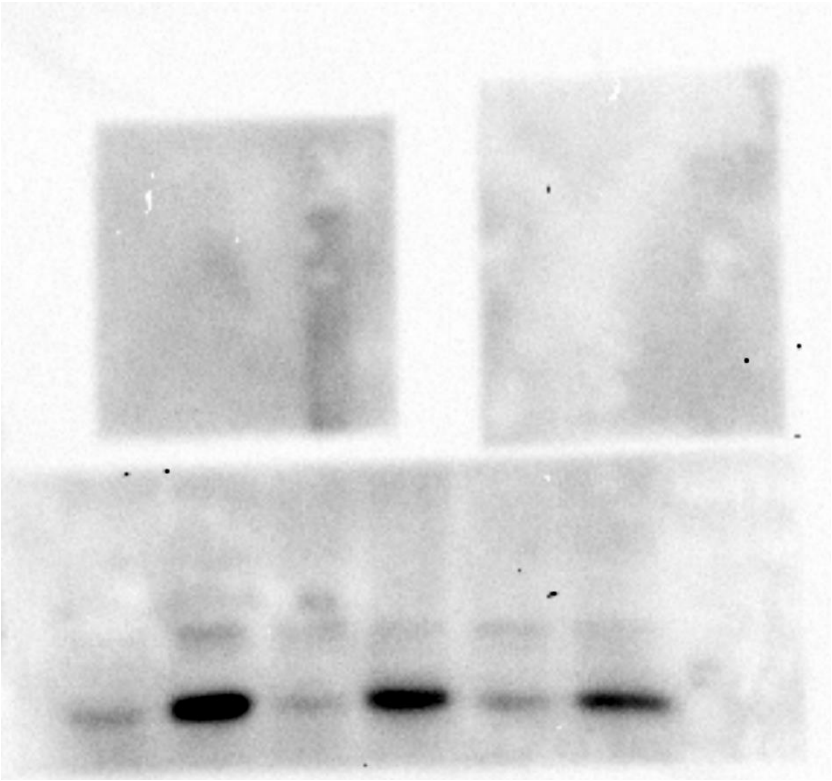

pAkt

|            |   |   |     |   |     |   |
|------------|---|---|-----|---|-----|---|
| INS        | - | + | -   | + | -   | + |
| BPA<br>1nM | - | - | +   | + | +   | + |
|            |   |   | 24h |   | 48h |   |

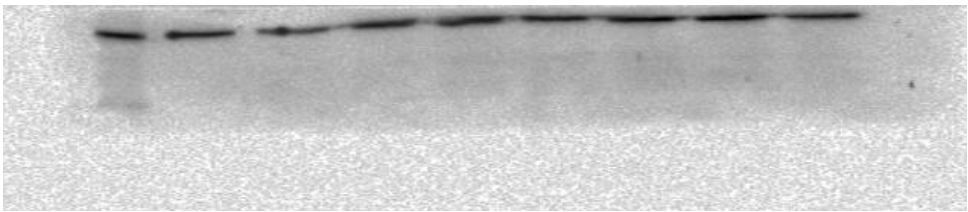

Akt

|     |   |   |     |   |     |   |
|-----|---|---|-----|---|-----|---|
| INS | - | + | -   | + | -   | + |
| BPA | - | - | +   | + | +   | + |
| 1nM |   |   |     |   |     |   |
|     |   |   | 24h |   | 48h |   |

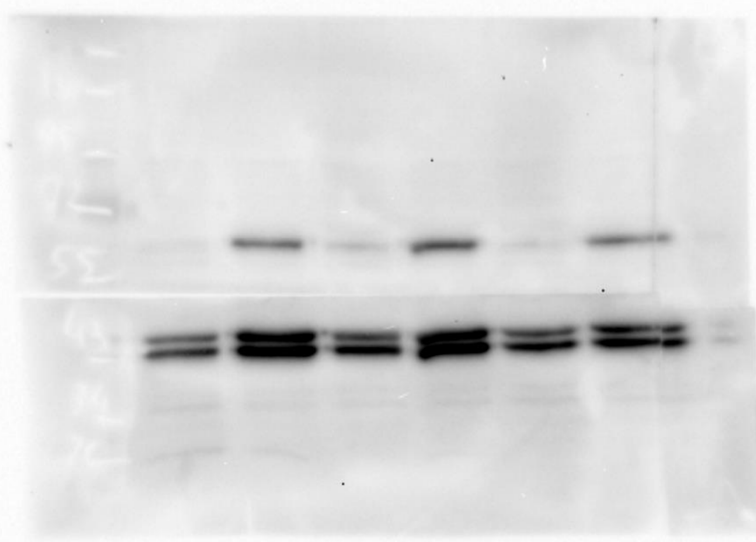

|            |   |   |     |   |     |   |
|------------|---|---|-----|---|-----|---|
| INS        | - | + | -   | + | -   | + |
| BPA<br>1nM | - | - | +   | + | +   | + |
|            |   |   | 24h |   | 48h |   |

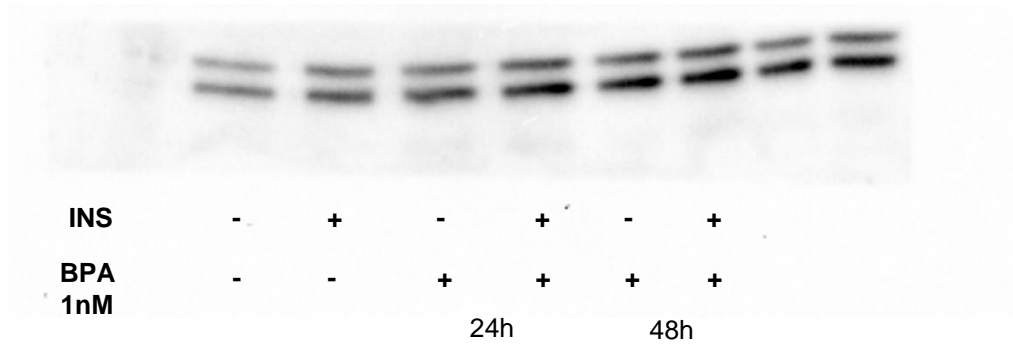

ERK

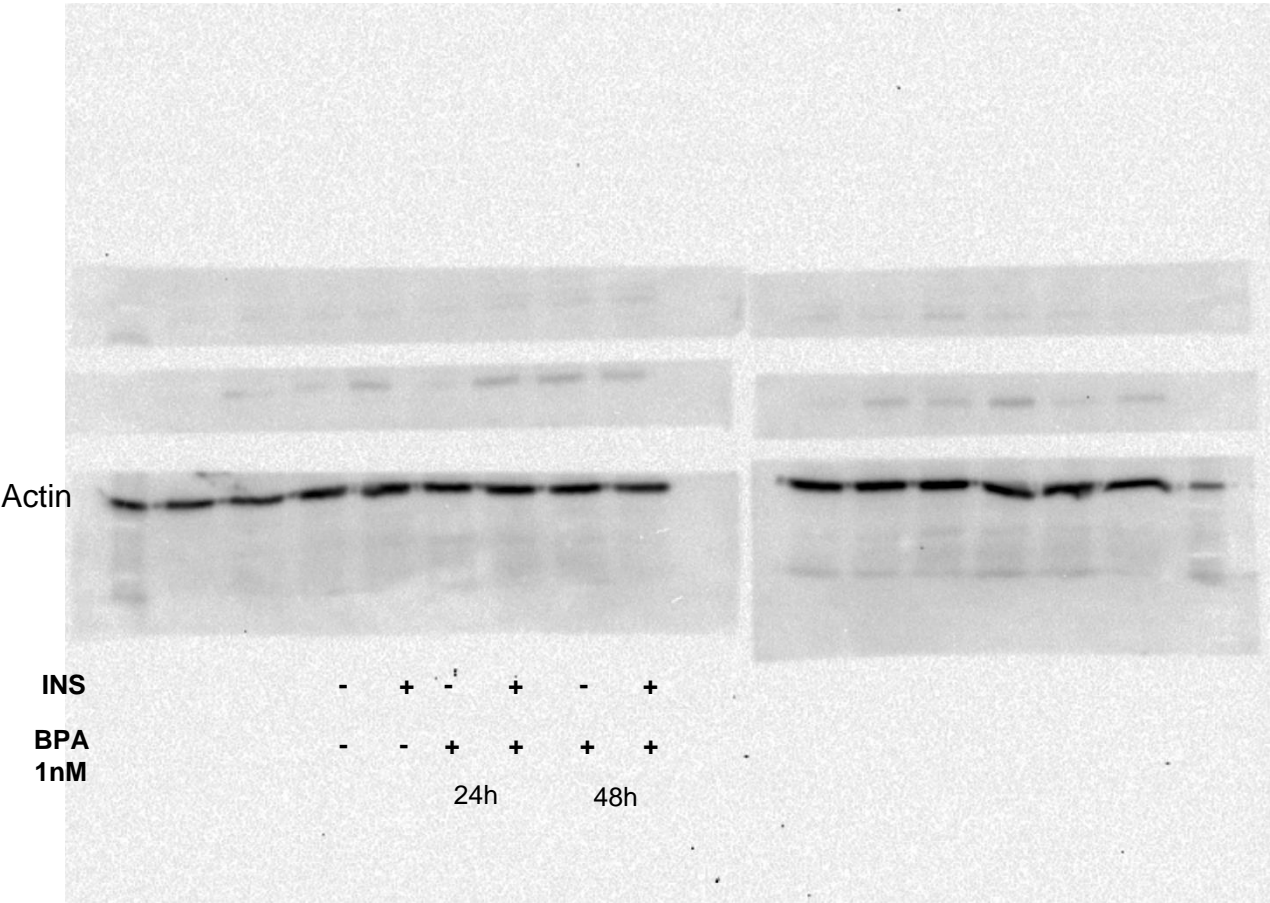

Supplement: S1 File — Original blot data supporting the results in Figure 3A. (PDF) [file pone.0264656.s001.pdf]

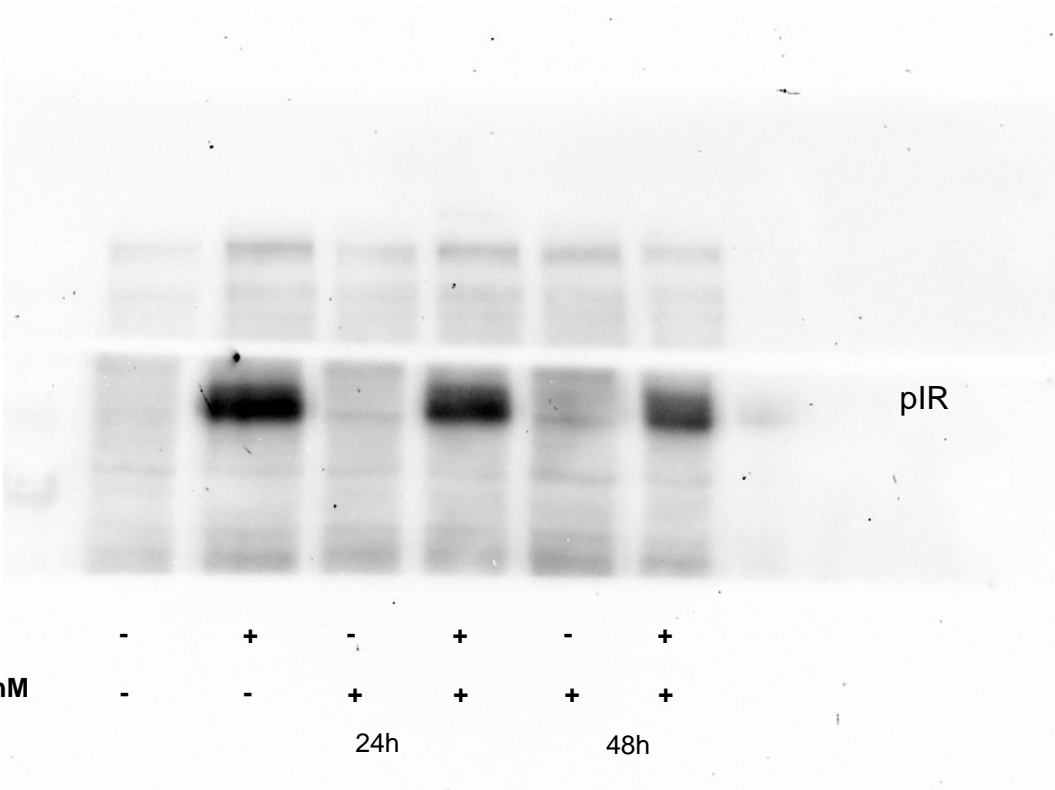

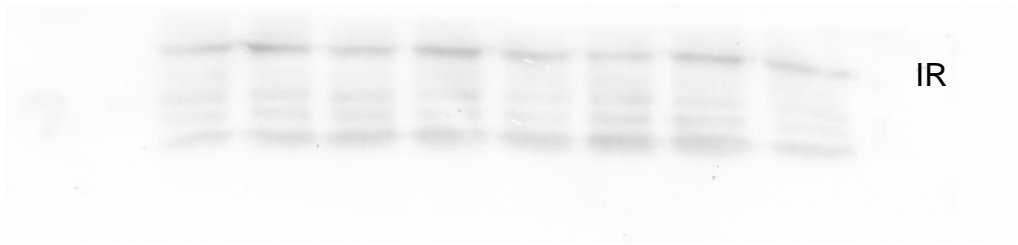

|            |   |   |     |   |     |   |
|------------|---|---|-----|---|-----|---|
| INS        | - | + | -   | + | -   | + |
| BPA<br>1nM | - | - | +   | + | +   | + |
|            |   |   | 24h |   | 48h |   |

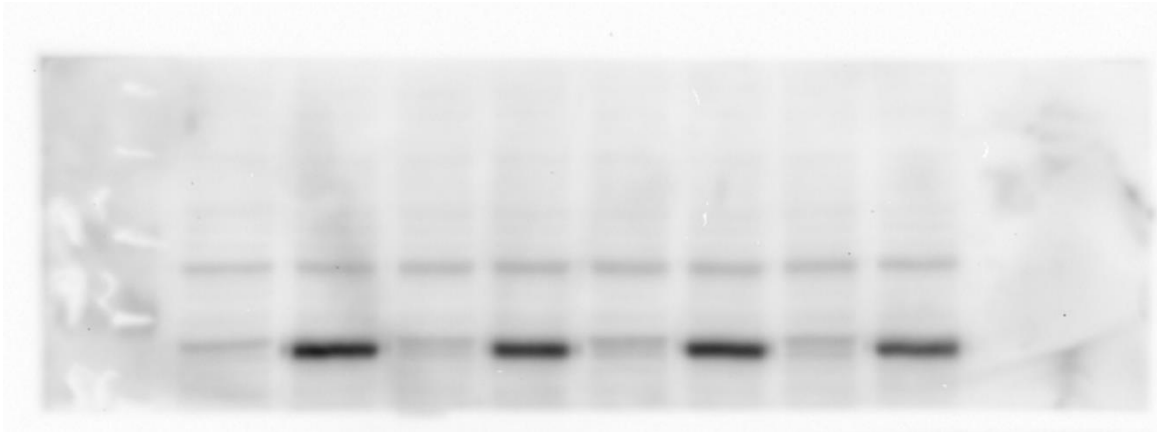

pAkt

|     |   |   |     |   |  |     |   |
|-----|---|---|-----|---|--|-----|---|
| INS | - | + | -   | + |  | -   | + |
| BPA | - | - | +   | + |  | +   | + |
| 1nM |   |   |     |   |  |     |   |
|     |   |   | 24h |   |  | 48h |   |

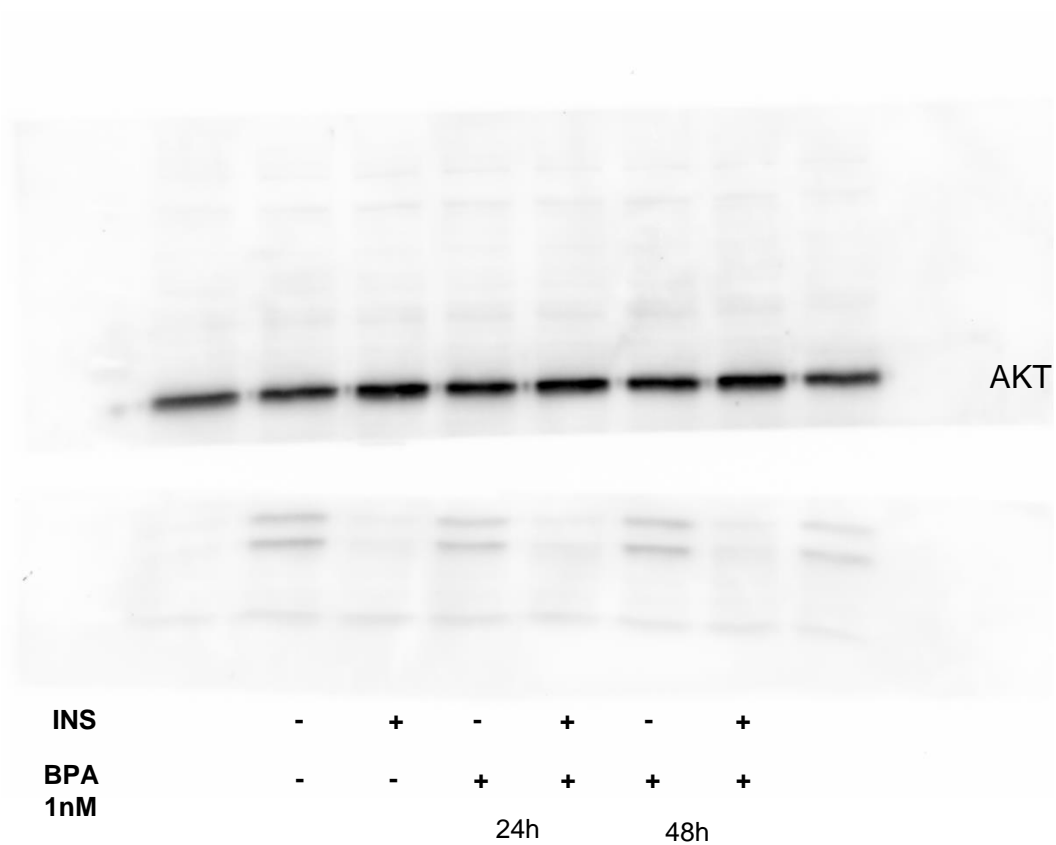

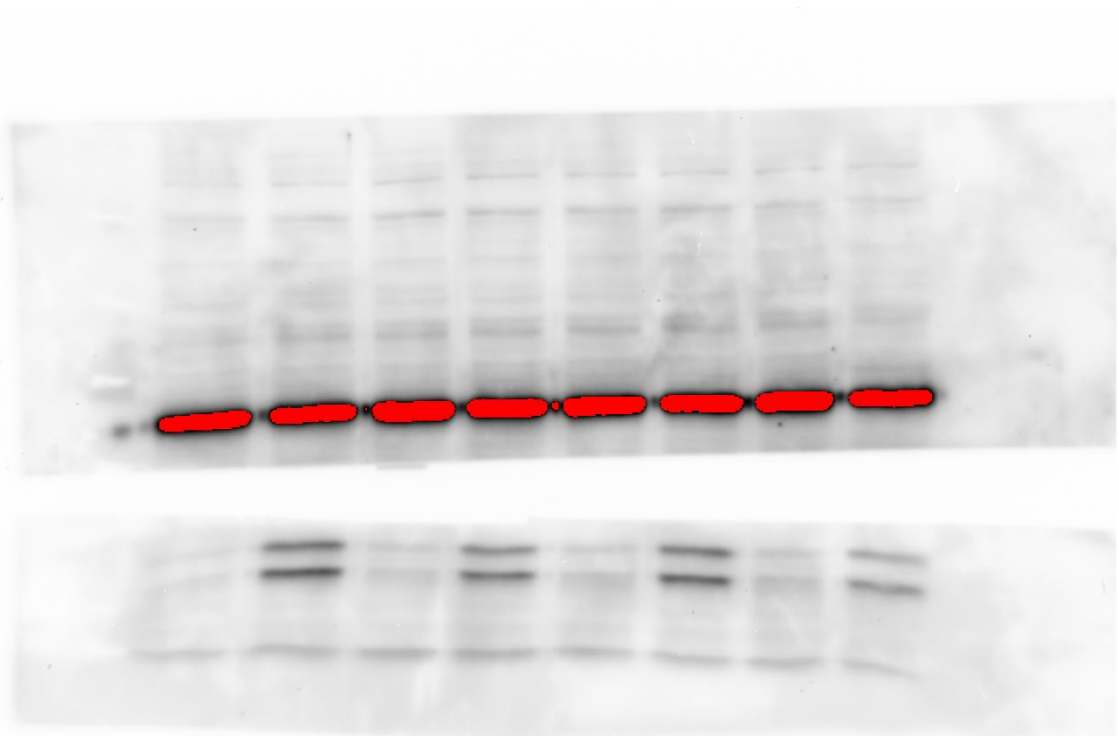

pERK

|     |   |   |     |   |  |  |     |   |
|-----|---|---|-----|---|--|--|-----|---|
| INS | - | + | -   | + |  |  | -   | + |
| BPA | - | - | +   | + |  |  | +   | + |
| 1nM |   |   |     |   |  |  |     |   |
|     |   |   | 24h |   |  |  | 48h |   |

ERK

|            |   |   |     |   |     |   |
|------------|---|---|-----|---|-----|---|
| INS        | - | + | -   | + | -   | + |
| BPA<br>1nM | - | - | +   | + | +   | + |
|            |   |   | 24h |   | 48h |   |

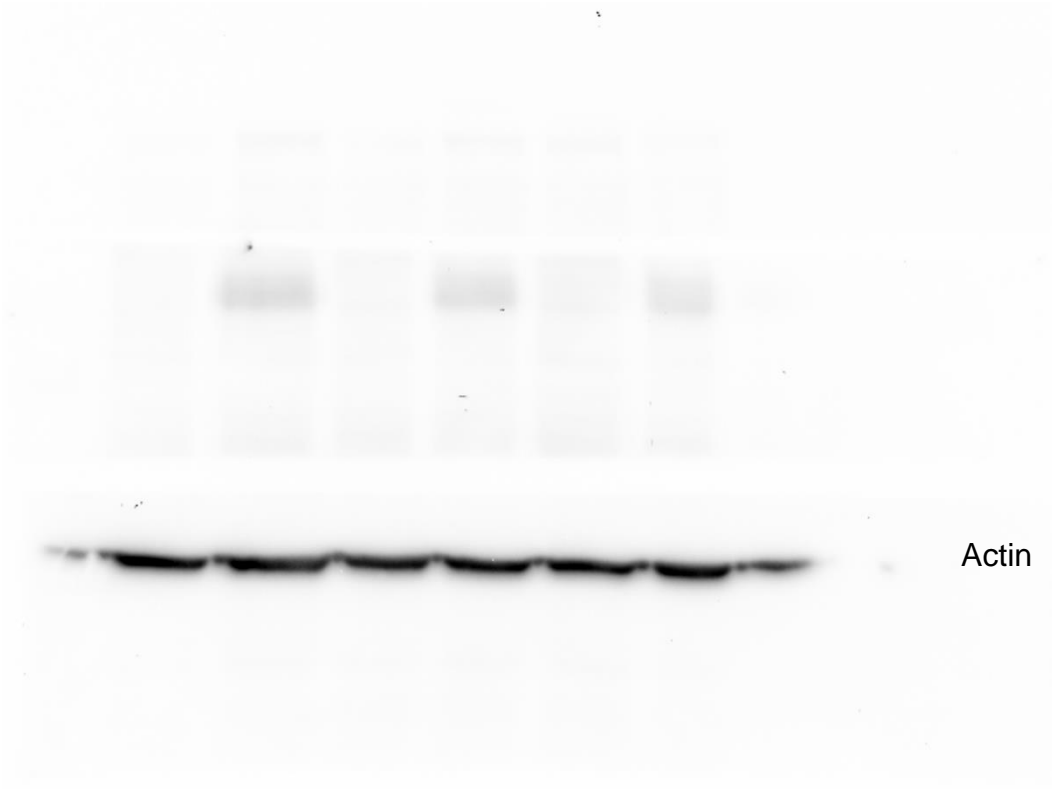

|         |   |   |     |   |     |   |
|---------|---|---|-----|---|-----|---|
| INS     | - | + | -   | + | -   | + |
| BPA 1nM | - | - | +   | + | +   | + |
|         |   |   | 24h |   | 48h |   |

Supplement: S2 File — Original blot data supporting the results in Figure 3C. (PDF) [file pone.0264656.s002.pdf]

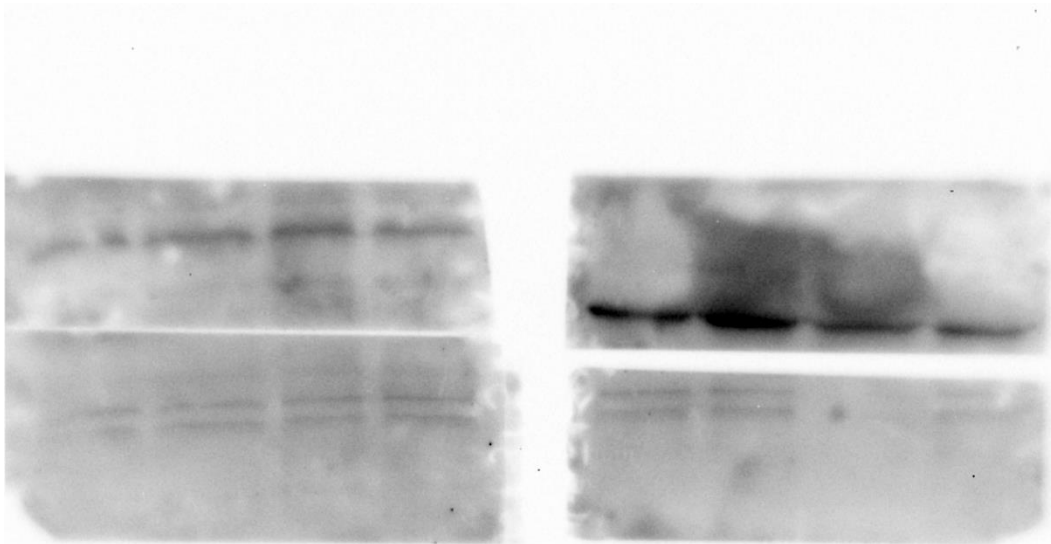

pJNK

|                    |   |   |   |
|--------------------|---|---|---|
| <b>BPA<br/>1nM</b> | - | + | + |
| <b>SP600125</b>    | - | - | + |

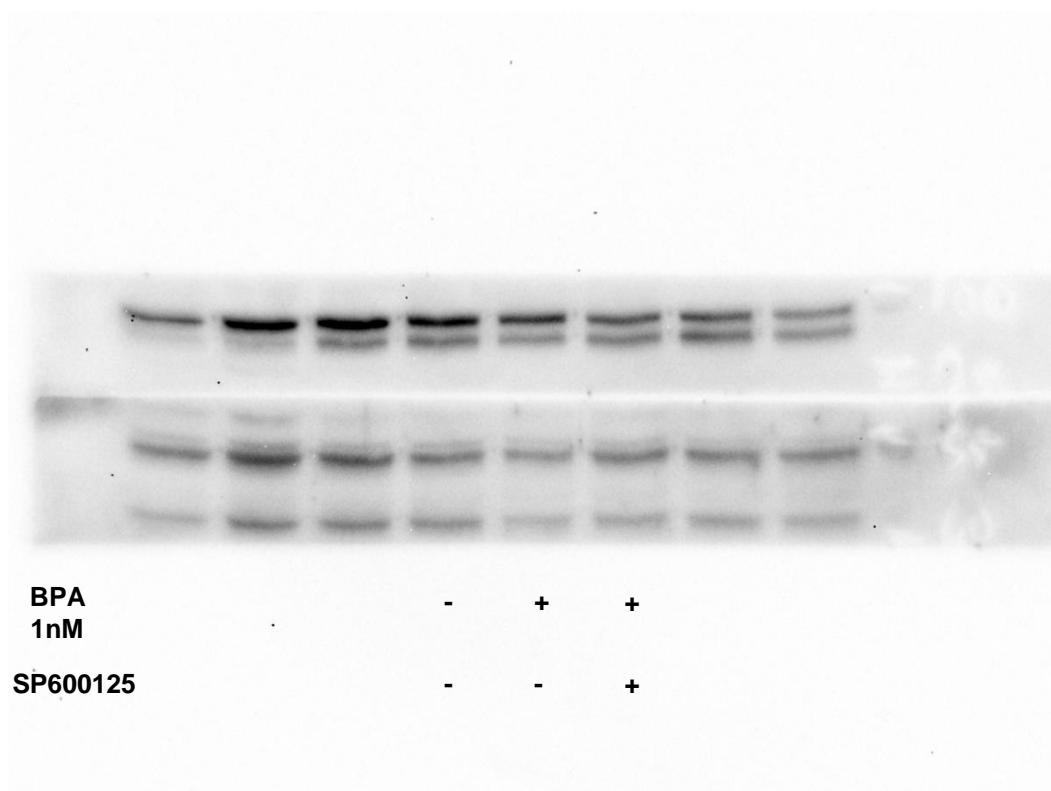

JNK

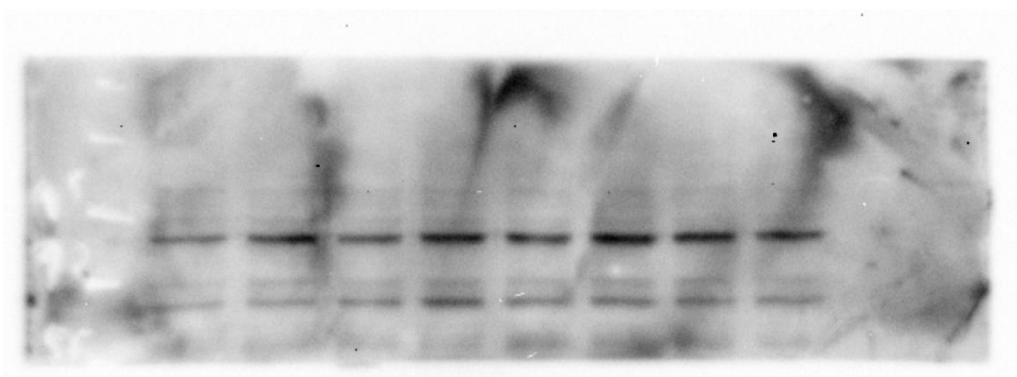

pSTAT3

|            |   |   |   |
|------------|---|---|---|
| BPA<br>1nM | - | + | + |
| SP600125   | - | - | + |

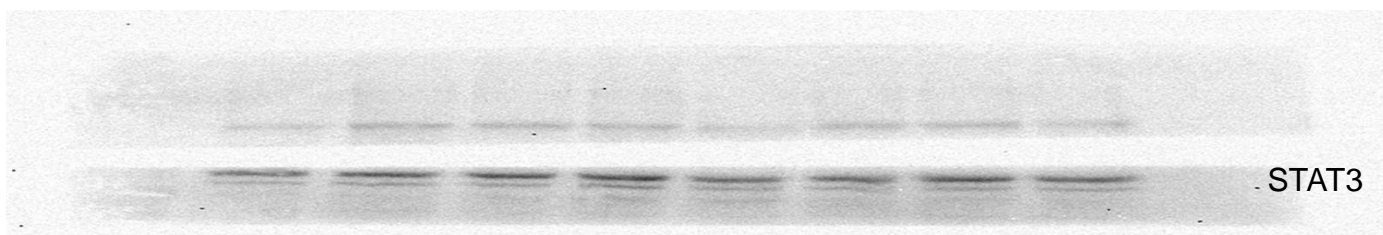

|                    |   |   |   |
|--------------------|---|---|---|
| <b>BPA<br/>1nM</b> | - | + | + |
| <b>SP600125</b>    | - | - | + |

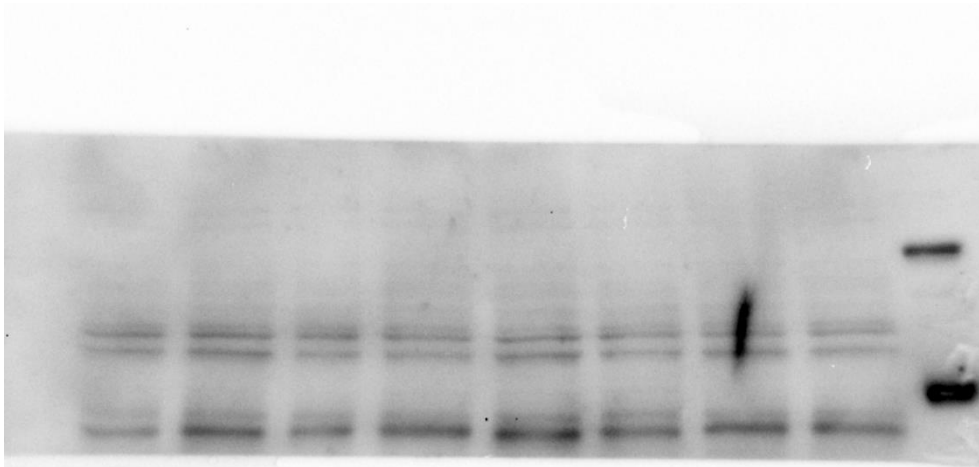

Actin

BPA  
1nM

- + +

SP600125

- - +

Supplement: S3 File — Original blot data supporting the results in Fig 7A. (PDF) [file pone.0264656.s003.pdf]

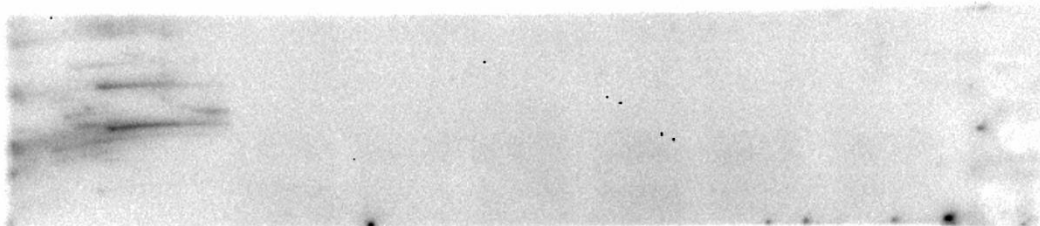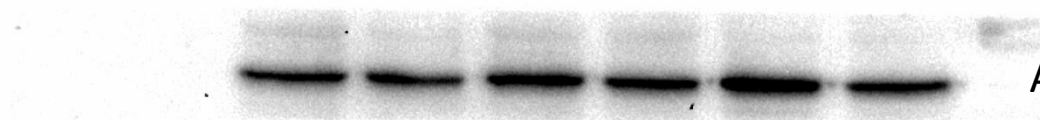

AKT 7c

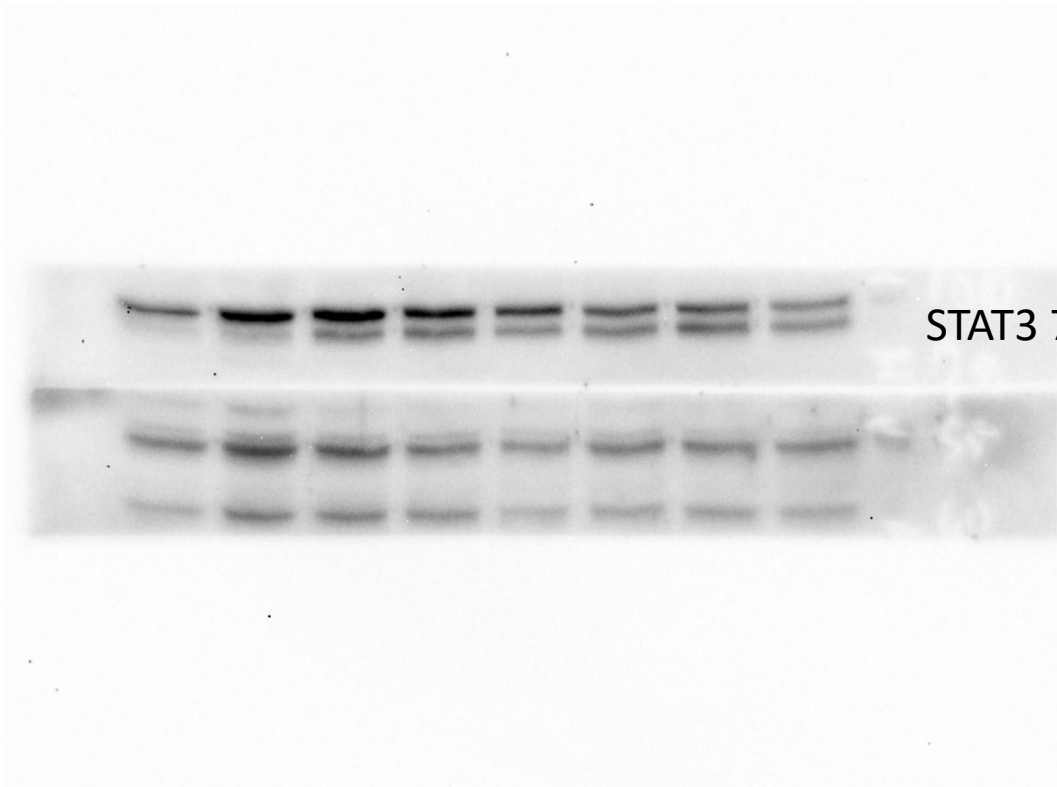

STAT3 7a

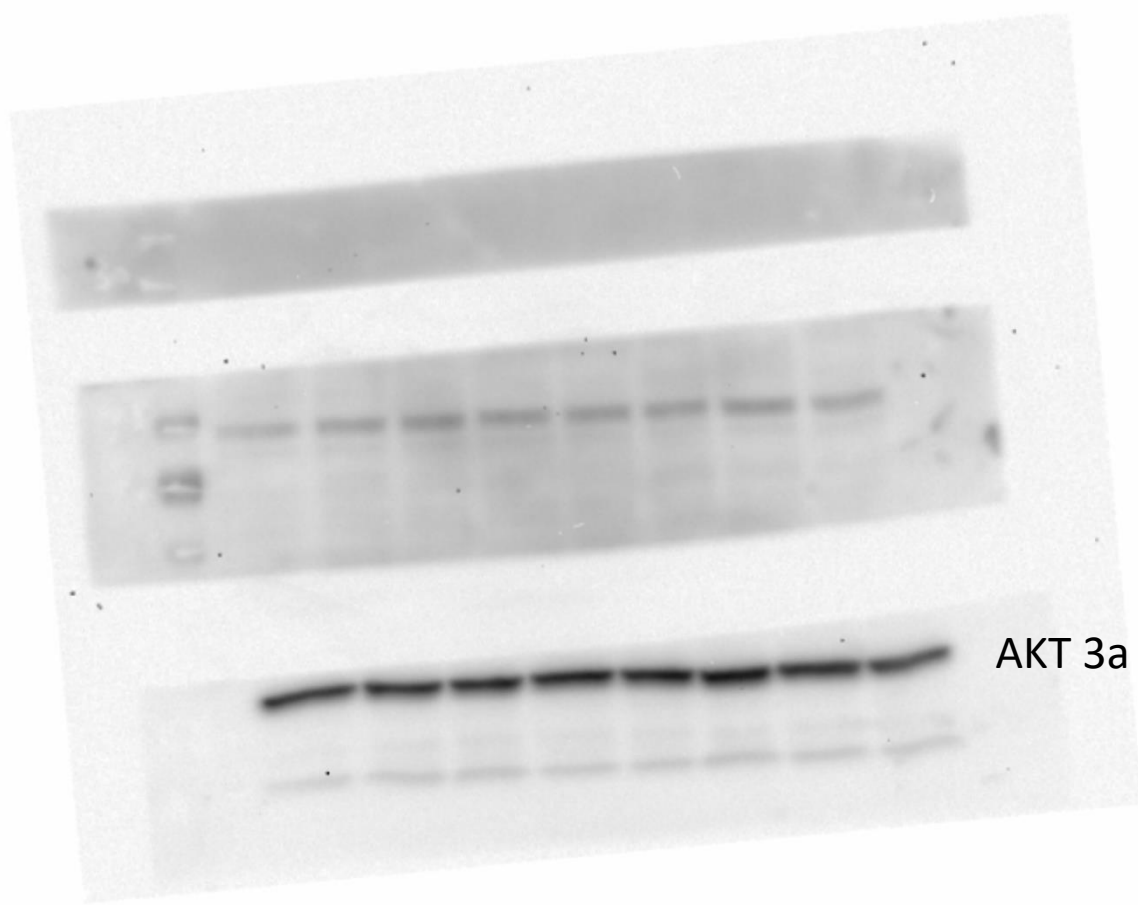

AKT 3a

Supplement: S5 File — Additional original blot data supporting the results in Figs 3 and 7. (PDF) [file pone.0264656.s005.pdf]
