# Supplementary material for: Correction: Bisphenol-A Impairs Insulin Action and Up-Regulates Inflammatory Pathways in Human Subcutaneous Adipocytes and 3T3-L1 Cells
Source: PLoS One. 2022 Feb 24;17(2):e0264656. doi: 10.1371/journal.pone.0264656 (PMC8870594; doi:10.1371/journal.pone.0264656)
Supplement: S4 File — Original blot data supporting the results in the corrected Fig 7C. (PDF) [file pone.0264656.s004.pdf]

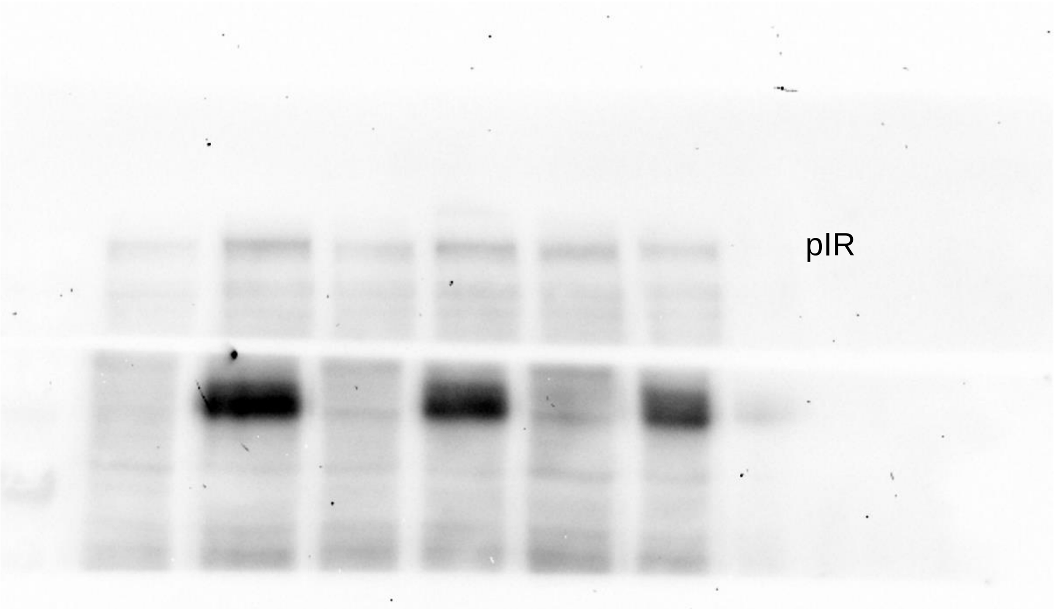

INS	-	+	+	+
BPA 1nM	-	-	+	+
SP600125	-	-	-	+

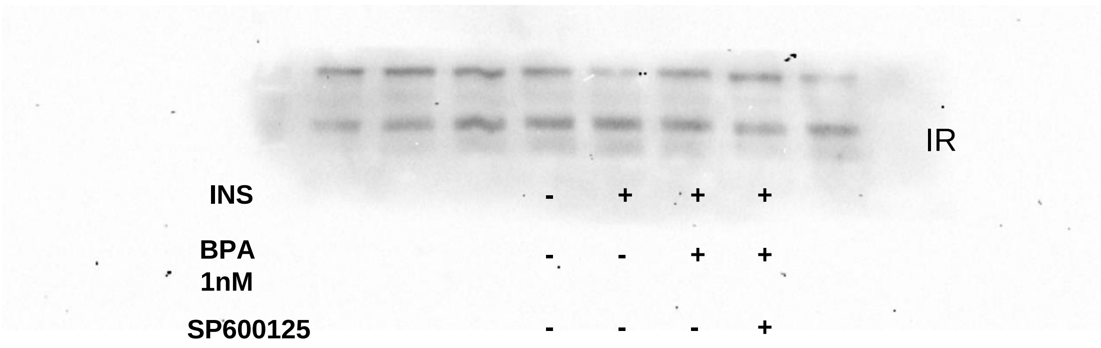
IR
INS - + + +
BPA 1nM - - + +
SP600125 - - - +

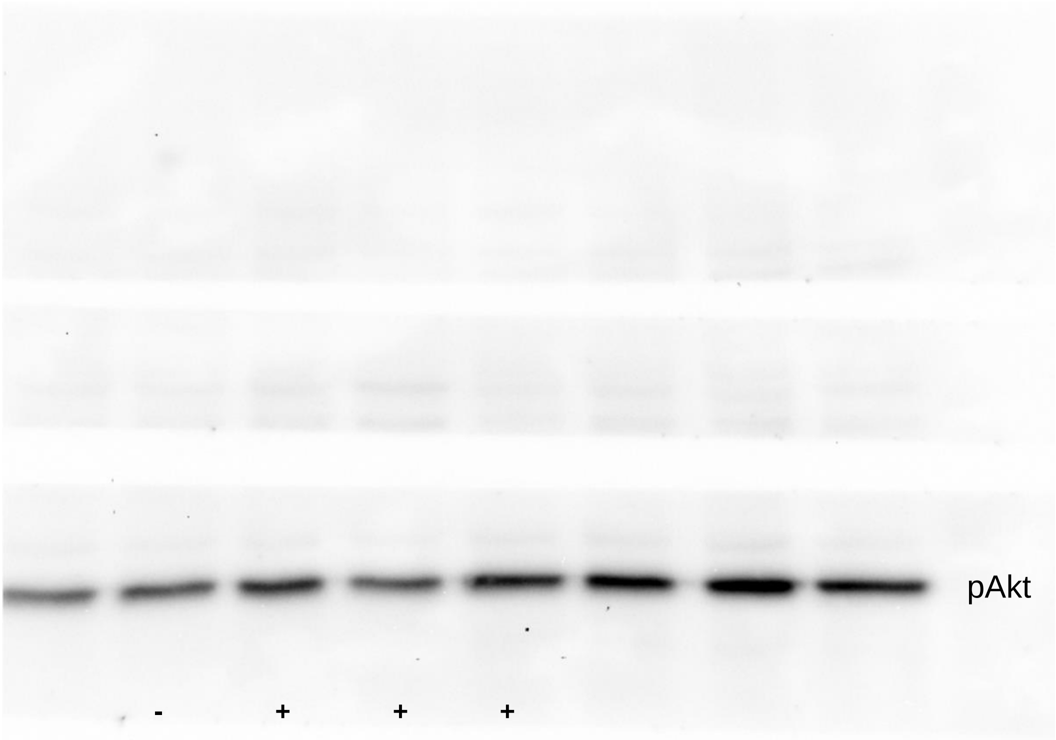

**INS**	-	+	+	+
**BPA 1nM**	-	-	+	+
**SP600125**	-	-	-	+

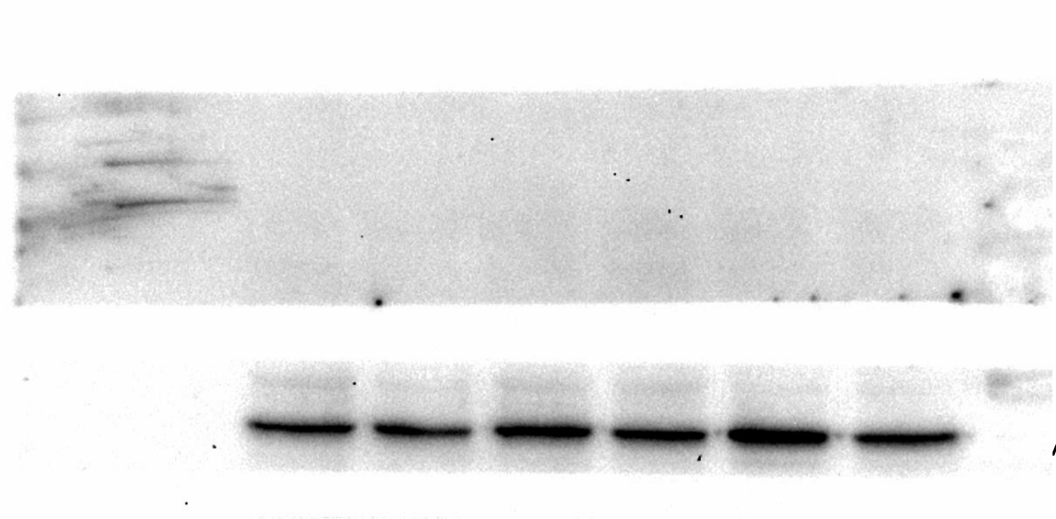

AKT

INS	-	+	+	+
BPA 1nM	-	-	+	+
SP600125	-	-	-	+

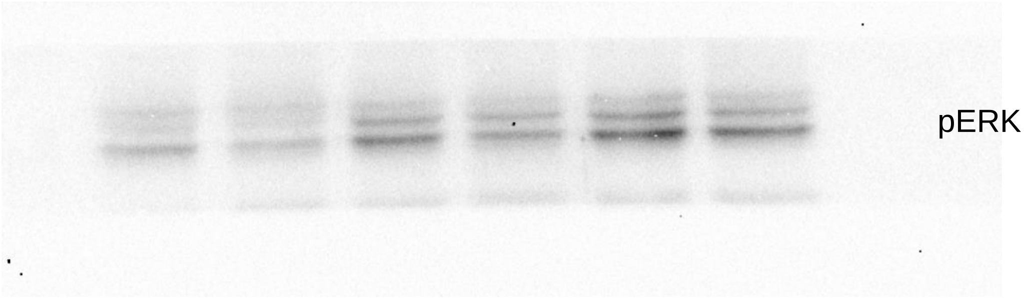

**INS**	**-**	**+**	**+**	**+**
**BPA 1nM**	**-**	**-**	**+**	**+**
**SP600125**	**-**	**-**	**-**	**+**

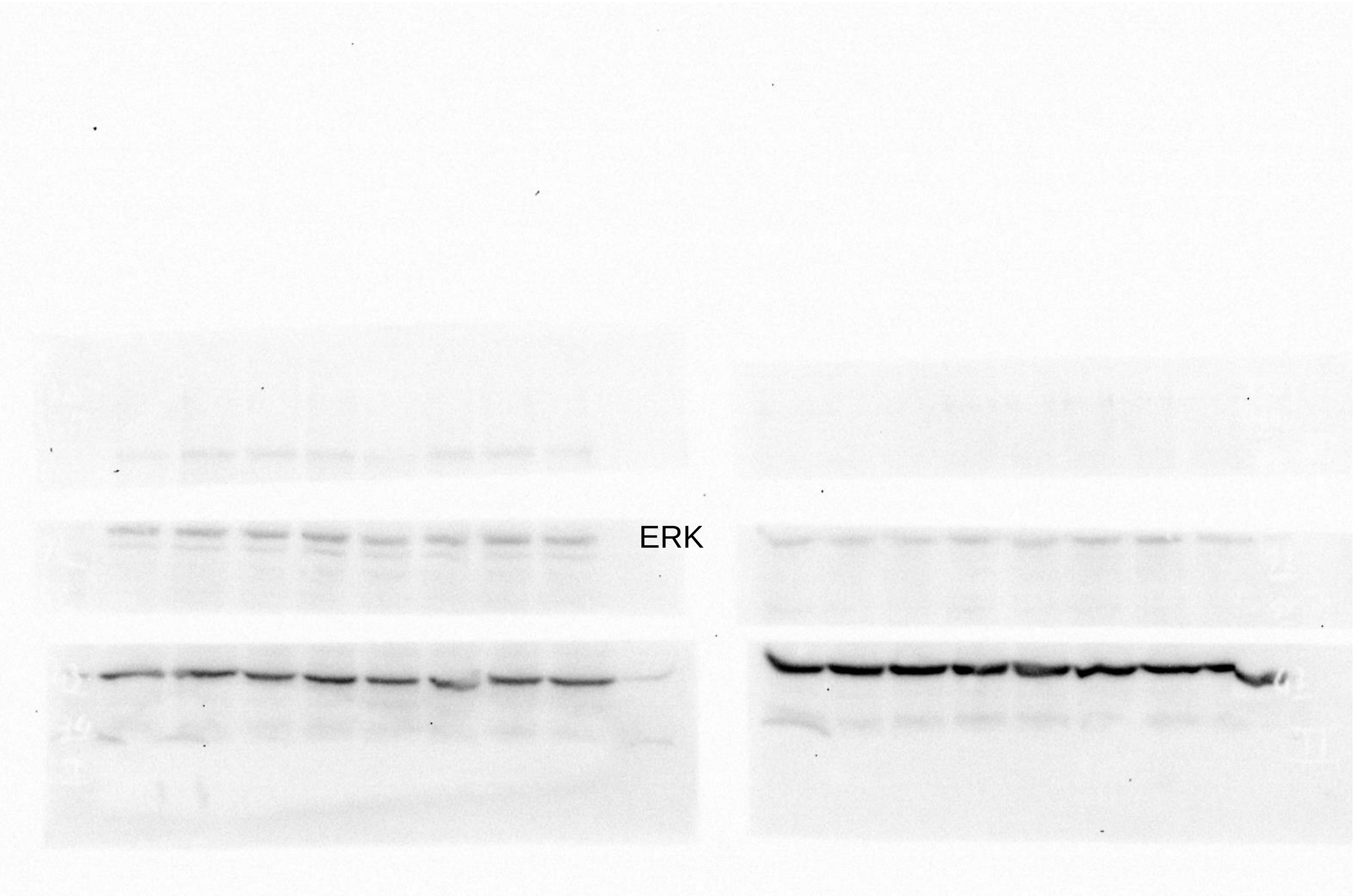

**INS**	-	+	+	+
**BPA 1nM**	-	-	+	+
**SP600125**	-	-	-	+

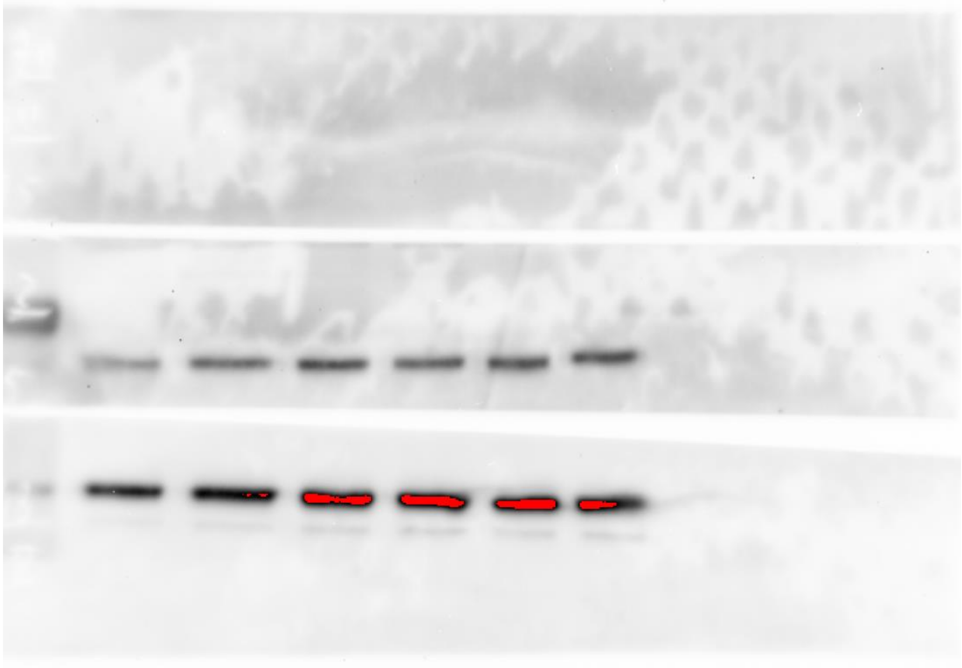

Actin

**INS**	**-**	**+**	**+**	**+**
**BPA 1nM**	**-**	**-**	**+**	**+**
**SP600125**	**-**	**-**	**-**	**+**
